# Neural Basis of Simple Arithmetic Processing in a Native and Second Language

**DOI:** 10.1162/NOL.a.278

**Published:** 2026-09-04

**Authors:** Vanessa R. Cerda, Macarena Suárez-Pellicioni, Nicole Y. Wicha, James R. Booth

**Affiliations:** Department of Psychology and Communication, Texas A&M International University, Laredo, TX, USA; Department of Educational Studies in Psychology, Research Methodology, and Counseling, University of Alabama, Tuscaloosa, AL, USA; Department of Neuroscience, Developmental and Regenerative Biology, University of Texas at San Antonio, San Antonio, TX, USA; Department of Psychology and Human Development, Vanderbilt University, Nashville, TN, USA

**Keywords:** arithmetic, bilingualism, fMRI, late learners, language bias, L2 expertise

## Abstract

Typically, bilinguals learn multiplication facts in their native language (L1), resulting in a performance advantage for arithmetic in L1 compared to a second language (L2). This performance advantage has been shown to be greater for individuals with lower L2 proficiency, suggesting that an individual’s language background may play a role in arithmetic processing. The current study aimed to determine whether adult learners of an L2 with limited L2 proficiency engage shared or separate cortical regions to process simple arithmetic across languages. Participants verified small (2 × 3) and large (8 × 9) multiplication problems in both languages, while functional magnetic resonance imaging (fMRI) was acquired. Small problems are thought to engage verbal memory to a greater extent than less practiced, large problems. Participants verified problems faster and more accurately in their L1 than L2. fMRI data suggest that the language differences in performance were not driven by differences in recruitment of the storage of arithmetic facts, as we did not observe expected differences in the engagement of the superior and middle temporal gyri (STG/MTG) across languages. Instead, these adults differentially engaged the left inferior frontal gyrus (IFG) across languages, suggesting that language differences in behavior may have been driven by their ability to engage more effortful arithmetic retrieval processes in L1, but not in L2. Additionally, participants exhibited expected differences in brain activation based on problem size, where small problems engaged STG/MTG and IFG associated with verbal retrieval processes and large problems presented with correct solutions engaged quantity processes associated with parietal regions.

## INTRODUCTION

Simple arithmetic facts, like multiplication tables, are typically learned through verbal rehearsal in the early years of schooling. Individuals who are bilingual often only learn and rehearse these facts in one of their two languages, and as a result, they typically prefer to perform simple arithmetic in that language (Dewaele, 2007; Vaid & Menon, 2000). Because of this phenomenon, multiplication facts are thought to be stored as language-specific representations in memory. In support of this idea, studies investigating bilingual arithmetic processing often report that bilinguals are faster and more accurate at solving simple arithmetic problems in the language they learned math in as compared to their other language, supporting a language bias for arithmetic (Cerda et al., 2019, 2023; Dehaene et al., 1999; Frenck-Mestre & Vaid, 1993; Lotus Lin et al., 2019; Marsh & Maki, 1976; Salillas & Wicha, 2012; Spelke & Tsivkin, 2001; Tamamaki, 1993; Van Rinsveld et al., 2016, 2017).

However, recent evidence suggests that a language bias for arithmetic is not always as robust for bilinguals who learned both languages early in development and have balanced language skills in adulthood (Cerda et al., 2022, 2023, 2024; Cerda & Wicha, 2023; Martinez-Lincoln et al., 2015). In fact, increased second language (L2) proficiency can mitigate language differences for arithmetic processing (Tamamaki, 1993). Similarly, individuals with lower L2 proficiency typically elicit larger differences in performance across languages on arithmetic and numeric tasks (see Garcia et al., 2021). Thus, an individual’s language background seems to play a role in processing simple arithmetic across a bilingual’s two languages (see Cerda & Wicha, 2023, for review). The goal of the current study was to determine whether adult learners of a L2 with limited L2 proficiency engage shared or separate cortical regions to process simple arithmetic in their native language (L1), which is also the language in which arithmetic was learned, and their later learned L2, in which they are less proficient.

The first study to suggest that relative language proficiency may impact arithmetic processing across languages was a behavioral study testing native speakers of Japanese who learned English at different ages in adulthood, resulting in varying proficiency in L2 across the sample (Tamamaki, 1993). When participants were asked to produce the solutions to multi-operand addition and subtraction problems in both of their languages, bilinguals with lower L2 proficiency produced solutions faster in their L1 than their L2. In contrast, bilinguals with high L2 proficiency produced solutions at comparable speeds across languages. Specifically, response times were faster in L1 for bilinguals with low L2 proficiency compared to high L2 proficiency bilinguals. Additionally, response times were faster in L2 for high L2 proficiency bilinguals compared to low L2 proficiency bilinguals. Tamamaki concluded that differences across languages were most likely attributed to encoding/response processes that were faster in L1 compared to L2, especially for bilinguals with less L2 experience. As bilinguals increase their use of L2, the ease of arithmetic processing in L2 increases, as well. This study shows that increased proficiency in a L2 leads to more efficient and comparable arithmetic performance across languages.

More recently, Garcia et al. (2021) performed a meta-analysis of 38 behavioral studies investigating arithmetic verification, calculation, and number naming. Their analysis revealed that bilinguals with lower L2 proficiency elicited larger differences in performance across languages on arithmetic and number tasks, which was driven by better performance in the language math was originally learned in compared to the other language. They also reported that when the L1 was also the language used for math learning, the language bias for arithmetic performance was even more pronounced. In line with Tamamaki’s findings, bilinguals with higher L2 proficiency tended to show comparable performance across languages for math tasks.

Math cognition models have attempted to explain performance differences for arithmetic across different number formats or languages. For example, the Triple Code Model (TCM) (Dehaene et al., 1999; Spelke & Tsivkin, 2001) suggests that because simple arithmetic facts are memorized via verbal rehearsal, they are stored as part of a learned lexicon of verbal associations in memory. As a result, the TCM proposes that, when problems are presented in other formats (e.g., Arabic digits), arithmetic facts must be transcoded into an auditory verbal code to be retrieved from memory. This model does not make explicit predictions about bilingualism, but by inference bilinguals should engage in direct retrieval of arithmetic facts in the language used for learning and use different processes, like translation, when those facts are presented in other languages (see Spelke & Tsivkin, 2001). Given that TCM does not address bilingualism directly, it is unclear how a bilingual’s language background, such as age of acquisition, proficiency, frequency and manner of use, etc., might influence processing.

The only model that directly addresses bilingual arithmetic is the Encoding Complex Model (ECM; Campbell & Clark, 1992; Campbell, 1994; Campbell et al., 1999; Campbell & Epp, 2004). The ECM is based on behavioral data from Chinese–English bilinguals and suggests that separate arithmetic memory representations exist for each format in which problems appear, including across a bilingual’s two languages. The ECM proposes that bilinguals access arithmetic facts from these language-specific representations, where retrieval efficiency is dependent on experience using the facts in each language. As an extension of the ECM, the Bilingual Encoding Complex Model (Bernardo, 2001) additionally proposed that factors like language proficiency or phonological length of number words may influence the efficiency of access to language-specific representations.

The relationship between factors related to a bilingual’s language background and the presence of a robust language bias for arithmetic has never been tested directly using brain measures, however there is evidence of a relationship in the bilingual arithmetic neuroimaging and electrophysiological literatures (see Cerda & Wicha, 2023, for a review). Across these studies, early bilinguals (i.e., bilinguals who learned both languages in early childhood) who are proficient in both languages typically do not show robust differences in these brain measures across their languages when doing arithmetic (Cerda et al., 2019, 2022, 2023, 2024), whereas bilinguals who learned an L2 later or were less fluent in their L2 showed more robust differences across languages when performing arithmetic (Lotus Lin et al., 2019; Venkatraman et al., 2006; Wang et al., 2007).

Three recent event-related potential studies carefully controlled for both language proficiency and age of language acquisition to study simple multiplication processing across languages in bilingual adults (Cerda et al., 2022, 2023) and children (Cerda et al., 2019). In these studies, the authors tested Spanish–English bilinguals who learned both languages before learning simple arithmetic and had balanced language proficiency. These bilinguals showed equivalent brain responses when verifying the correctness of simple multiplication problems presented in both of their languages. It was only under more demanding task parameters (i.e., speeded presentation of arithmetic problems) that the bilingual adults showed subtle differences in their brain responses (Cerda et al., 2023). Specifically, under these increased task demands, more difficult problems (i.e., problems with larger numerical solutions) were harder to categorize or produce in the language not used for original learning of arithmetic facts. The findings suggest that language differences were not driven by access to the facts from memory. Similarly, a parallel functional magnetic resonance imaging (fMRI) study in Spanish–English bilingual adults did not find evidence that proficient bilinguals differentially engaged brain regions involved in arithmetic processing (Cerda et al., 2024). Together, these findings suggest that bilinguals who have strong proficiency and who learned both languages before learning arithmetic engage the same neurocognitive processes when verifying simple multiplication facts in both of their languages.

To our knowledge, no neuroimaging study has directly addressed whether factors of a bilingual’s language background, like L2 proficiency, influence arithmetic verification. Previous studies suggest that later L2 learning and limited L2 proficiency might drive larger differences in the cortical regions engaged for arithmetic across languages (see Cerda & Wicha, 2023, for review). For example, Venkatraman et al. (2006) tested English–Chinese bilinguals with self-reported English dominance. Participants were trained on novel base-7 addition problems in only one language (i.e., the trained language). When tested on these problems, bilingual participants were overall more accurate at solving base-7 problems in the trained language compared to the untrained language. They also engaged brain regions thought to be involved in phonological memory, including the bilateral frontal regions and the left inferior parietal lobule, to a greater extent in the untrained language compared to the trained language. The authors took this to suggest that trained facts are stored in the language of training and this representation is accessed, potentially via translation, when participants are presented with the same problems in an untrained language. Similarly, Wang et al. (2007) tested Chinese–English bilinguals who learned English after the age of 12. Participants were asked to verify the correctness of arithmetic problems that are not usually explicitly memorized (i.e., addition and multiplication problems involving a two-digit operand) in their L1 and L2. L2 engaged a more extensive network of cortical regions than L1, including the left inferior frontal gyrus (IFG). The authors again suggested engagement of this frontal area may result from translation from L2 to L1.

The current study aimed to directly investigate the effects of late L2 learning and low L2 proficiency on simple multiplication processing. We tested native English speakers who gained limited Spanish proficiency as an L2 later in life, long after learning simple multiplication facts in English during childhood. Participants were asked to verify the correctness of single-digit multiplication problems presented with auditory number word operands in both English and Spanish and an Arabic Digit solution. The verification task was identical to a previous study with early, balanced bilingual adults (Cerda et al., 2024). We focused on simple multiplication facts as they are typically learned through rote memorization and should promote engagement of direct verbal retrieval processes, at least in the L1 (language of learning the arithmetic facts).

In line with a previous study on bilingual arithmetic (Cerda et al., 2024), multiplication problems varied in problem difficulty (i.e., problem size). The problem size effect is a well-known phenomenon in the math cognition literature (see Zbrodoff & Logan, 2005, for a review), where small problems (e.g., 2 × 3) are typically more practiced and are retrieved more efficiently than large problems (e.g., 8 × 9). As a result, large problems are more likely to promote the engagement of back-up strategies like calculation, transformation, or decomposition. Previous work has found interactions between the format in which a problem is presented and the problem size effect in both monolinguals (visual operands vs. auditory operands, Dickson & Wicha, 2019) and bilinguals (English auditory operands vs. Spanish auditory operands, Cerda et al., 2023), where less familiar formats promoted less efficient processing particularly for larger, less practiced problems. This interaction should in principle be even more robust across languages (formats) for late learners of an L2. That is, larger more difficult problems should reveal larger differences in behavior and cortical representation across languages compared to smaller problems, as detailed below.

Previous neuroimaging studies in monolinguals have established specific brain regions that are differentially engaged based on problem size. Temporal regions, including the left superior and middle temporal gyri (STG/MTG), are involved in the storage of arithmetic facts in verbal memory, especially when they have been explicitly memorized (Prado et al., 2013). Increased activity in temporal regions has been associated with children’s increased expertise in arithmetic facts, particularly for more practiced small problems, reflecting the strengthening of semantic associations between operands and their solutions as children receive more years of math instruction (Prado et al., 2014). In contrast to small problems, fronto-parietal regions are more strongly involved in solving large problems, reflecting the reliance on more effortful processes (De Smedt et al., 2011). Specifically, the left IFG is more active during the retrieval of less practiced facts, such as large multiplication problems, even in adults (Prado et al., 2011). Additionally, activation in the intraparietal sulcus (IPS) has been suggested to reflect the involvement of quantity-based processes to solve large problems (De Smedt et al., 2011; Prado et al., 2013; Suárez-Pellicioni et al., 2018).

In line with this, early, balanced adult bilinguals engage the right IPS to a greater extent when processing large multiplication problems than small problems and the left STG/MTG to a greater extent for small than large problems, regardless of the language of presentation (i.e., spoken number words, Cerda et al., 2024). Contrary to previous studies (De Smedt et al., 2011; Prado et al., 2011), the sample of bilingual adults in Cerda et al. (2024) also showed greater engagement of left IFG for small problems than large problems, suggesting effortful retrieval even for more practiced problems. Critically, there were no differences in engagement in any of these regions across languages in this bilingual sample.

The current study measures behavior and brain activation patterns during multiplication verification for problems presented in English and Spanish to native English-speaking adults who learned Spanish as an L2 after learning arithmetic (i.e., late bilinguals). Given previous findings, we test three hypotheses:

1) A main effect of Language will be measurable for each of our regions of interest (ROIs; left STG/MTG, left IFG, and bilateral IPS). Given that STG/MTG are associated with the verbal representation of math facts, adult L2 learners should engage STG/MTG to a greater degree when verifying arithmetic in English, their L1 and language for learning arithmetic, than in the L2, Spanish. Conversely, the L2 will recruit IFG and IPS, which have been associated with more effortful retrieval and reliance on quantity processes, respectively, to a greater degree than the L1.
2) There will be a main effect of Problem Size for each of the ROIs. Based on the Problem Size effect patterns observed for early, balanced adult bilinguals (Cerda et al., 2024) we expect STG/MTG activation for small problems and IPS activation for large problems. This would suggest that late learners have strong verbal representations of small multiplication facts in memory but must engage quantity-processing regions for larger problems. We may also see IFG activation modulated by Problem Size, but it is unclear whether this area would be engaged to a greater extent for small problems (as found by Cerda et al., 2024, using an identical task) or for large problems (De Smedt et al., 2011; Prado et al., 2011).
3) There will be a Problem Size by Language interaction. Specifically, L1 will engage STG/MTG to a greater extent than L2 and this effect will be most apparent for more frequently encountered small problems, which rely more on access to verbal representations. However, we hypothesized that L2 will recruit IFG and IPS to a greater extent than L1, with the effect being greater for large problems, which are harder to retrieve and more associated with reliance on quantity-processing and more effortful mechanisms.

## METHODS

The study hypotheses and analytical plan were preregistered through Open Science Framework prior to beginning data analyses (see https://osf.io/wfgvk). The analysis plan aligns with a previous study on bilingual arithmetic processing (Cerda et al., 2024), which included a sample of early Spanish–English balanced bilingual adults performing the same experimental tasks herein. Additionally, each exploratory analysis was preregistered prior to conducting any subsequent analyses.

### Participants

The final sample consisted of 32 participants (21 female; ages 18–31; average age: 21.5) recruited from Vanderbilt University. Informed written consent was obtained from all participants with oversight from the Institutional Review Boards of both Vanderbilt University and the University of Texas at San Antonio (via institutional agreement). Three additional participants were excluded from the final sample (one due to failure to complete all experimental tasks and two due to excessive Spanish exposure based on the criteria outlined below).

All participants were native English speakers who learned multiplication in English and reported using English as their current preferred language for math. Three participants reported Korean, Mandarin, or Ewe as a simultaneous L1; however, all participants learned Spanish as an L2 through formal school instruction after 10 years of age (average age of acquisition: 13 years). Participants did not take any advanced Spanish classes (i.e., upper-division Spanish courses) and did not have Spanish exposure at home (e.g., no Spanish-speaking parents or grandparents) aside from general exposure to basic Spanish vocabulary through children’s Spanish television shows, music with Spanish lyrics, or Spanish-speaking individuals in their community. Participants reported speaking English a majority of the time (average at home: 94.5% English, 0.22% Spanish; average at school/work: 97.0% English, 1.8% Spanish), where limited time speaking Spanish was dedicated to practicing Spanish through coursework/homework or engaging with Spanish media (e.g., music or TV). Additionally, participants did not have any Spanish immersion experiences greater than 2 weeks. On average, participants self-rated their English-speaking and comprehension ability at 7, using a Likert scale with 1 indicating “beginner” and 7 indicating “native speaker”. Conversely, participants rated their Spanish speaking ability on the same scale at 2.9 (range: 2–4) and comprehension ability at 3.1 (range: 1–5). Besides these self-reported measures, we confirmed that all participants had very limited Spanish proficiency using standardized measures (see below).

Participants were right-handed (assessed by the Edinburgh Inventory, Oldfield, 1971; range 0.35–1.00), had normal or corrected-to-normal vision, normal hearing, no history of cognitive or perceptual deficits, were not diagnosed with language delays or learning disabilities, and were not taking medication that affected their cognition at the time of testing.

### Standardized Measures

Participants’ language proficiency in English was determined using the Picture Vocabulary (Test 14) and the Oral Comprehension (Test 15) subtests of the Woodcock-Johnson Tests of Achievement (Woodcock et al., 2001). Parallel versions of these tests were administered in Spanish from the Batería III Woodcock-Muñoz (Muñoz-Sandoval et al., 2005). These tests required participants to demonstrate vocabulary and oral language comprehension in each language. These measures have been used to characterize language abilities in previous investigations of arithmetic processing in Spanish–English bilinguals with balanced proficiency (see Cerda et al., 2019, 2022, 2023, 2024).

**Figure 1.**
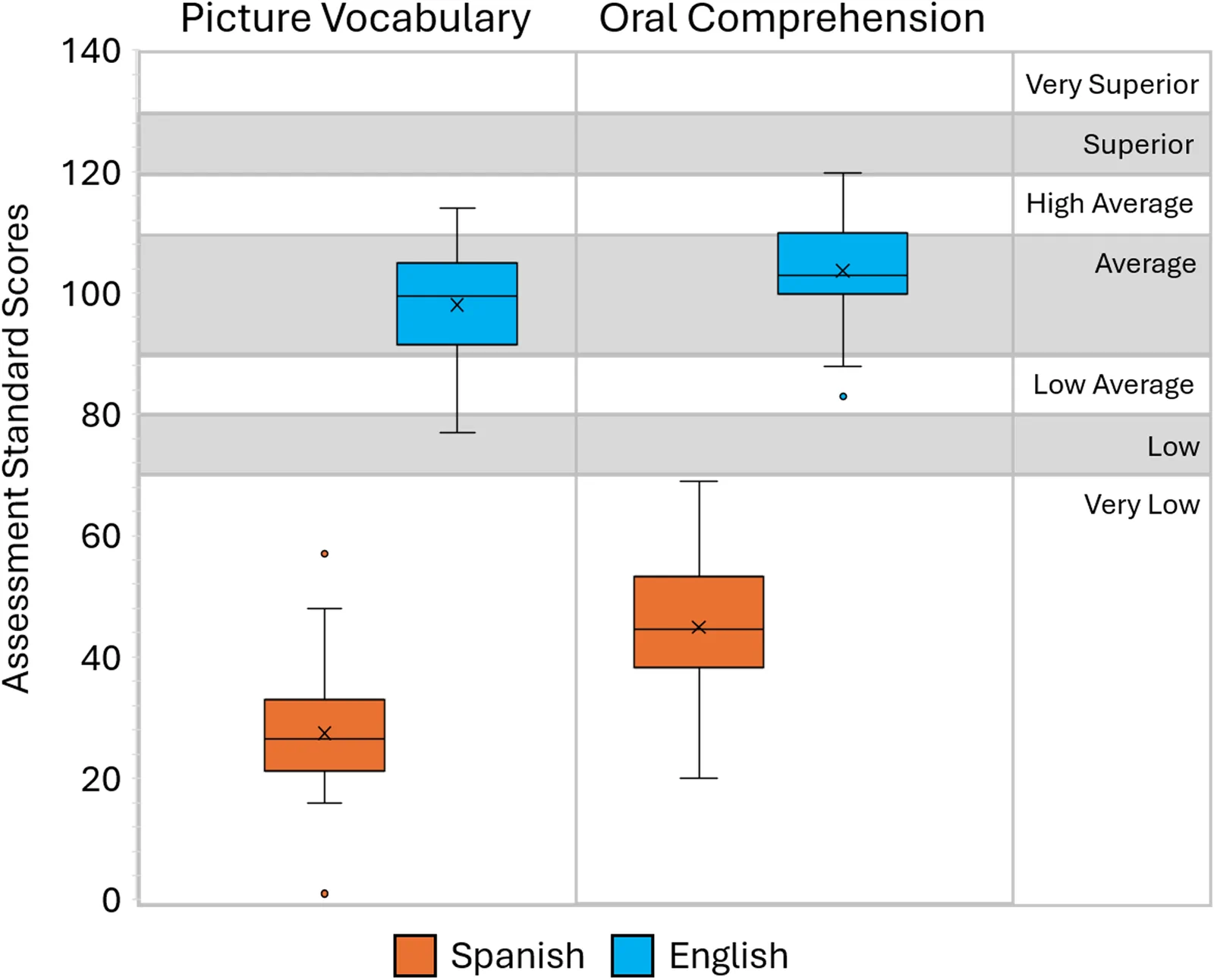
Distribution of standard scores for language assessments in Spanish and English. Standard scores for Spanish assessments are depicted in orange box plots and English scores are depicted in blue box plots. The size of the boxes represents the interquartile range, the × in the center of the boxes represents the mean, solid bars within the boxes represent the median, and the whiskers represent the maximum and minimum values excluding outliers. Outliers are defined as any value exceeding the third quartile by 1.5 times the interquartile range and are indicated by orange and blue dots on the plot. The shaded regions delineate the Woodcock-Johnson Proficiency Battery Classifications, defined by age-based standard scores.

Aged-normalized standard scores and corresponding proficiency classifications were obtained from the Woodcock-Johnson Proficiency Battery (WJPB-R) based on participants’ raw assessment scores in each language. Participants all scored at the “very low” WJPB-R proficiency level in the two subtests in Spanish, where standard scores were below 69 for both the Spanish Picture Vocabulary and Oral Comprehension subtests (see Figure 1). All participants scored at the “low average” WJPB-R proficiency level or above in English (apart from three participants who were classified as having “low” proficiency in English Picture Vocabulary). Critically, all participants were strongly dominant in English over Spanish.

Multiplication fluency was measured using the Math Fluency Multiplication subtest of the Wechsler Individual Achievement Test (Wechsler, 1992). This subtest comprises 40 single-digit multiplication problems and asks participants to answer as many problems as possible in 60 s. Participants’ age-based standardized scores ranged from 83 to 123, where six participants had low average multiplication skills, 14 had average multiplication skills, nine had superior multiplication skills, and three had high average multiplication skills. As mentioned above, all participants learned simple arithmetic, and all subsequent basic math concepts in English. However, all subjects reported knowing their numbers at least up to 10 in Spanish. Measures of working memory and phonological awareness were collected but are outside the scope of the current research questions.

### Scanner Tasks

Participants performed three tasks in a single experimental session in the following order: a single-digit multiplication task, a verbal localizer task, and a quantity localizer task. The multiplication and verbal localizer tasks were administered in separate runs for Spanish and English with language-specific instructions (see Grosjean, 1998), counterbalancing language order across participants. Language order assignments for each participant were the same for both the multiplication and verbal localizer tasks. Each task was completed in the order specified above with one language presented directly after the other (e.g., Spanish multiplication task, English multiplication task, Spanish verbal localizer, and English verbal localizer). The verbal task was used as a functional localizer for temporal-frontal areas in each language, whereas the quantity task was used as a functional localizer of quantity processing regions in parietal cortex. Each task also included control trials intermixed with the experimental trials in which a blue square was presented, and participants were asked to press a response button with their index finger when the box turned red. Identical tasks were previously used in a study of proficient, early bilinguals (Cerda et al., 2024).

#### Single-digit multiplication verification task

**Figure 2.**
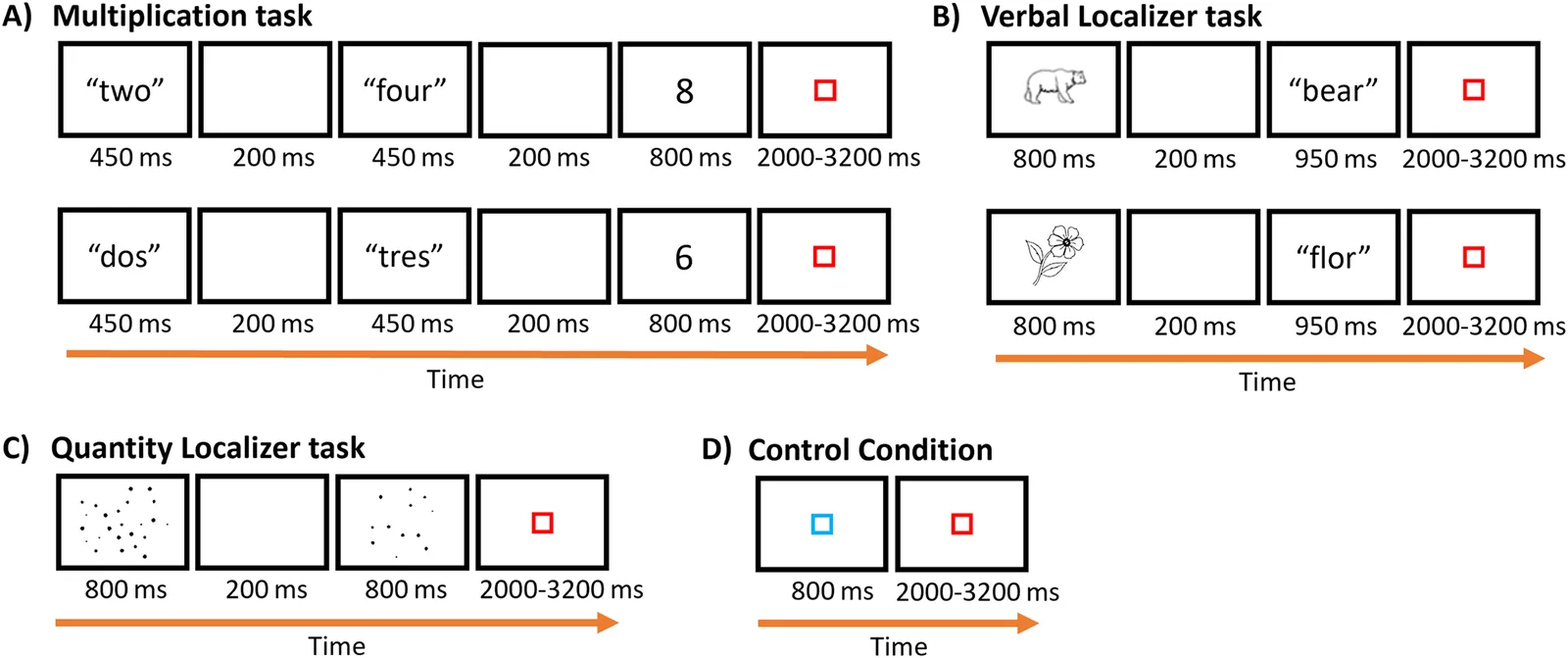
Sample trials across all experimental tasks. (A) Sample trial of the multiplication task in English (top) and Spanish (bottom). Participants did not see any written words. All auditory number words were normalized to 450 ms in duration. (B) Sample trial of the picture-word verification task (i.e., verbal localizer) in English (top) and Spanish (bottom). (C) Sample trial of the dot comparison task (i.e., quantity localizer). (D) Sample control trial used across all tasks.

Participants were presented with two runs of single-digit multiplication problems in English and two runs in Spanish (see sample trials in Figure 2A). For this task, participants heard two spoken number words, followed by a visual Arabic digit, and were asked to verify whether the Arabic digit was the correct product of the two spoken numbers or not (i.e., true vs. false). This paradigm was adapted from (Cerda et al., 2019) where spoken number words were used for problem operands as a way of engaging the participants in a specific language when performing the multiplication task. Proposed solutions were presented as an Arabic digit for all trials. As such, any effects at the solution could be attributed to the language used to access the math facts from memory rather than differences in processing the language of the solutions, per se. Visual stimuli were projected onto a screen that was viewed by the participants through a mirror attached to the MRI head-coil, and the audio was presented using MRI-compatible over-the-ear headphones. Behavioral responses were recorded using an MR-compatible button box. Participants used their right index finger if the proposed solution was true and their right middle finger if the proposed solution was false.

Thirty-six single-digit multiplication problems were presented per run and were classified based on problem size. In line with previous research, problems with true solutions smaller than 25 were considered small problems and problems with true solutions larger than 25 were considered large problems (see Cerda et al., 2023, 2024; Dickson & Wicha, 2019; Dickson et al., 2022). In each run, there were 12 small problems with true solutions, 12 large problems with true solutions, 6 small problems with false solutions, and 6 large problems with false solutions. To ensure equivalent task difficulty across languages, identical sets of core multiplication problems were presented in English and Spanish, using a different, semi-randomized order of trials.

An additional 12 control trials were included per run to account for basic visual stimulation and motor activity. For these trials, participants saw a blue square and were instructed to press a button with their index finger when the blue square turned red (see Figure 2D). With the inclusion of these trials, the duration of each run was approximately 6 min.

#### Verbal localizer—Picture-word verification task

Participants completed two runs of a picture-word verification task in English and two runs in Spanish. Language order assignments for each participant were the same as the language order of the multiplication task (e.g., starting both tasks in Spanish). During the task, participants saw a black-and-white line drawing of an object followed by a spoken word and determined whether the word matched the presented picture or not (see sample trials in Figure 2B). Behavioral responses were also recorded using an MR-compatible button box. Participants used their right index finger if the picture-word pairs matched and their right middle finger if they did not match. Forty experimental trials were presented per run, with an equal number of matched and mismatched trials. Mismatched trials were different across languages to avoid predictability, and words were matched on frequency and animacy across languages and conditions (see Cerda et al., 2019, for complete details on stimulus construction). Additionally, 13 control trials (identical to the multiplication task) were included per run (see Figure 2D). The duration of each run was approximately 6 min.

#### Quantity localizer—Dot comparison task

Participants completed two runs of the dot comparison task and were tasked with determining which of two sequentially presented dot arrays contained more dots (Figure 2C). This task has been used previously as a localizer of quantity regions in parietal cortex in monolinguals (Berteletti et al., 2014; Prado et al., 2014; Suárez-Pellicioni & Booth, 2018; Suárez-Pellicioni et al., 2020) and bilinguals (Cerda et al., 2024). Thirty-six experimental trials were divided as follows: 12 easy comparisons (i.e., a ratio of 1:3; e.g., 12 dots to 36 dots), 12 medium comparisons (i.e., a ratio of 1:2; e.g., 18 dots to 36 dots), and 12 hard comparisons (i.e., a ratio of 2:3; e.g., 24 dots to 36 dots). Dot size was controlled across arrays to ensure that participants were judging differences in the number of dots, rather than dot size or the total surface area covered by the dots. Participants used their right index finger if the first dot array contained more dots and their right middle finger if the second dot array contained more dots. Additionally, 12 control trials (Figure 2D), identical to the control trials included in the multiplication task, were presented per run. The duration of each run was approximately 3 min.

#### Stimulus timing

For the multiplication task, all spoken number words in both English and Spanish were normalized to 450 ms duration, which was chosen specifically to reduce distortion in both languages. Experimental trials began with the presentation of the first operand (450 ms duration) followed by a 200 ms interstimulus interval before the second operand (450 ms duration). During the presentation of the spoken operands, participants saw a blank white screen. Then 200 ms after the offset of the second operand, an Arabic Digit solution appeared on the screen for 800 ms. All problems were followed by variable periods of fixation (2,000 ms, 2,600 ms, or 3,200 ms; 1,200 ms jitter) to help with convolution of each trial, during which a red fixation square was presented. Participants were instructed to answer as quickly and accurately as possible as soon as they knew the answer and could respond until the beginning of the next trial. Each run ended with 22 s of passive visual fixation to also aid in convolution of the final trials.

Stimulus timing was similar for both localizer tasks. Trials began with the presentation of the first stimulus (i.e., line drawing or first dot array, depending on the task) for 800 ms, followed by a blank screen for 200 ms. For the verbal localizer, the second stimulus was a spoken word in English or Spanish, across separate blocks, presented for the natural duration of the spoken word up to 930 ms (English average word length: 645 ms; range: 410–930 ms; Spanish average word length: 643 ms; range: 390–930 ms) For the quantity localizer, the second stimulus was the second dot array, presented for 800 ms. For both tasks, the second stimulus was followed by variable periods of fixation (2,000 ms, 2,600 ms, or 3,200 ms; 1,200 ms jitter) to help with convolution of each trial, during which a red fixation square was presented. Participants were instructed to answer as quickly and accurately as possible as soon as they knew the answer and could respond until the beginning of the next trial. Each run ended with 22 s of passive visual fixation for convolution of the final trials.

### fMRI Data Acquisition

Images were collected using a Philips Elition 3.0T scanner at the Vanderbilt University Institute of Imaging Science Center for Human Imaging. The fMRI blood oxygenation level dependent signal was measured with a susceptibility weighted single-shot echo planar imaging (EPI) sequence. Functional images were acquired with multiband EPI, using interleaved slice ordering from foot to head. The following parameters were used: TE = 30 ms, flip angle = 68 s, matrix size = 256 × 256, field of view = 256, slice thickness = 2 mm, number of slices = 64, TR = 1,250 ms, voxel size: 2 × 2 × 2 mm, and a multiband acceleration factor of 4. Before functional image acquisition, a high resolution T1 weighted 3D structural image was acquired for each participant with the following parameters: TR = 1,900 ms, TE = 2.98 ms, matrix size = 256 × 256, field of view = 256 mm, slice thickness = 1 mm, number of slices = 192.

### fMRI Data Analysis

#### Preprocessing

Preprocessing and analysis of the fMRI data were computed using Statistical Parametric Mapping (SPM12; https://www.fil.ion.ucl.ac.uk/spm). First, all functional images were realigned to their mean functional image across runs. An anatomical brain mask was created by combining the segmentation products (i.e., grey matter, white matter, and cerebrospinal fluid) and then applied to its original anatomical image to produce a skull-stripped anatomical image. Then, the mean functional image and all functional images were co-registered to the skull-stripped anatomical image. After normalization, all the functional images were normalized to the standard T1 Montreal Neurological Institute (MNI) template. Slice timing correction was not applied, as the images were acquired with multiband EPI. Afterward, smoothing was applied to all the functional images with a 6 mm isotropic Gaussian kernel. All coordinates are reported in MNI space for consistency with previous fMRI studies of math cognition (e.g., Berteletti et al., 2014; Cerda et al., 2024; Prado et al., 2011, 2013, 2014; Suárez-Pellicioni et al., 2020; Suárez-Pellicioni & Booth, 2022).

To reduce movement effects on the brain signal, Art-Repair (https://cibsr.stanford.edu/tools/human-brain-project/artrepair-software.html) was used to identify outlier volumes. Outliers were defined as volume-to-volume head movement exceeding 1.5 mm in any direction, head movement greater than 5 mm in any direction from the mean functional image across runs, or deviations of more than 4% from the mean global signal intensity. Outlier volumes were repaired by interpolation between the nearest non-outlier volumes. Participants included in the analysis had no more than 10% of the total volumes repaired and no more than 6 consecutive volumes repaired in each run. Six motion parameters estimated during realignment were entered in the first-level modeling as regressors and the repaired volumes were deweighted (Mazaika et al., 2009).

To be included in the analyses, all participants performed higher than chance (i.e., 50%) verifying small problems in the multiplication task in English. Additionally, they had no response bias in either the multiplication or the picture-word verification task in English, defined as no greater than 50% accuracy difference between true and false problems in the multiplication task, or matched and mismatched trials in the picture-word task.

#### fMRI processing

Event-related statistical analysis was performed according to the general linear model (GLM). Activation was modeled as epochs with onset times time-locked to the presentation of the first stimulus in each trial (i.e., first spoken operand of the multiplication problem, line drawing of the verbal localizer, and first dot array in the numerosity localizer). To equate for power in the analysis, all proposed solutions (i.e., true and false) and all participants’ responses (i.e., accurate and inaccurate) were included in the model. All epochs were convolved with a canonical hemodynamic response function. The time series data were high-pass filtered (1/128 Hz), and serial correlations were corrected using an autoregressive AR model.

#### ROIs

Regions of interest were constructed by constraining brain activation from the two localizer tasks within anatomically defined regions in the left STG/MTG, the left IFG, and the bilateral intraparietal sulci (IPS). These anatomical regions were defined using the automated anatomical labeling (AAL) template (part of the Wfupickatlas tool). Template regions of left STG and left MTG were combined into a single anatomical mask. The IFG anatomical mask included the left pars opercularis, left pars triangularis, and left pars orbitalis. The anatomical mask for IPS was constructed by dilating (3D dilation of 2) both the inferior and superior parietal lobules and isolating the intersection between the two regions, similar to Suárez-Pellicioni and Booth (2018) and Cerda et al. (2024). This method was used to create separate ROIs in the left and right IPS.

**Figure 3.**
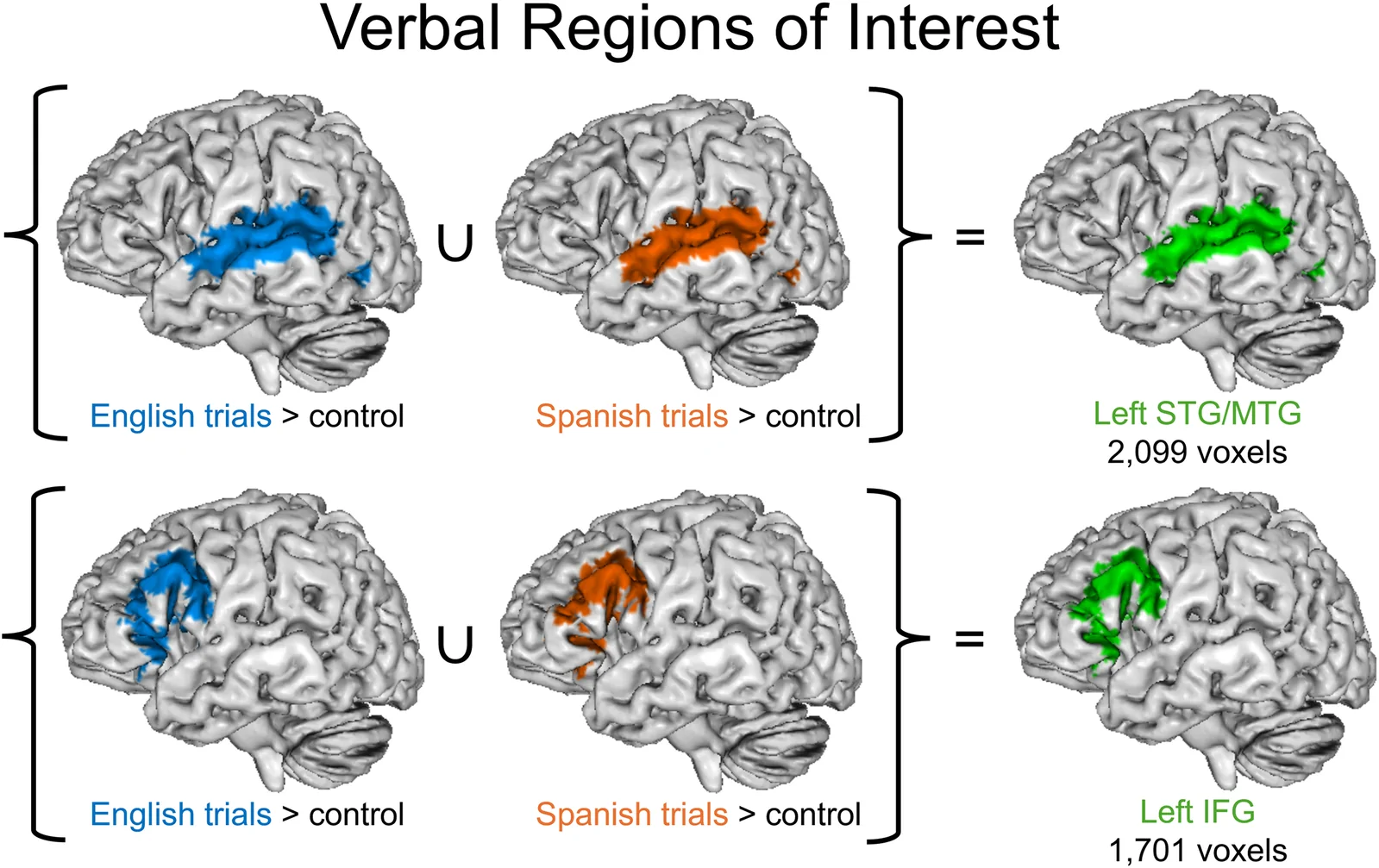
Regions of interest identified using the verbal localizer task. 25% of voxels showing maximal activation (regardless of significance) within left STG/MTG (top panel) and within left IFG (bottom panel) for the contrast of English trials greater than control (shown in blue in the left column) and the contrast of Spanish trials greater than control (shown in orange in the middle column). The right column shows, in green, the union of the extracted voxels across the two languages in temporal (top panel) and frontal (bottom panel) cortices.

To define combined (i.e., functional and anatomically defined) ROIs, these anatomical regions were used to constrain the brain activity from the localizer tasks. More specifically, verbal combined ROIs were defined by constraining brain activation during the verbal localizer tasks separately for each language (i.e., “all picture-word pairs in Spanish > control” and “all picture-word pairs in English > control” contrasts) within the left STG/MTG and left IFG. We then extracted the top 25% of voxels showing maximal activation (regardless of significance) for English (clusters in blue in Figure 3) and Spanish (clusters in orange in Figure 3). The union of the top voxels across the two languages constituted our combined verbal ROI (clusters in green in Figure 3). The resulting left MTG/STG and left IFG ROIs comprised 2,099 voxels (289 non-overlapping voxels across languages) and 1,701 voxels (387 non-overlapping voxels across languages), respectively.

**Figure 4.**
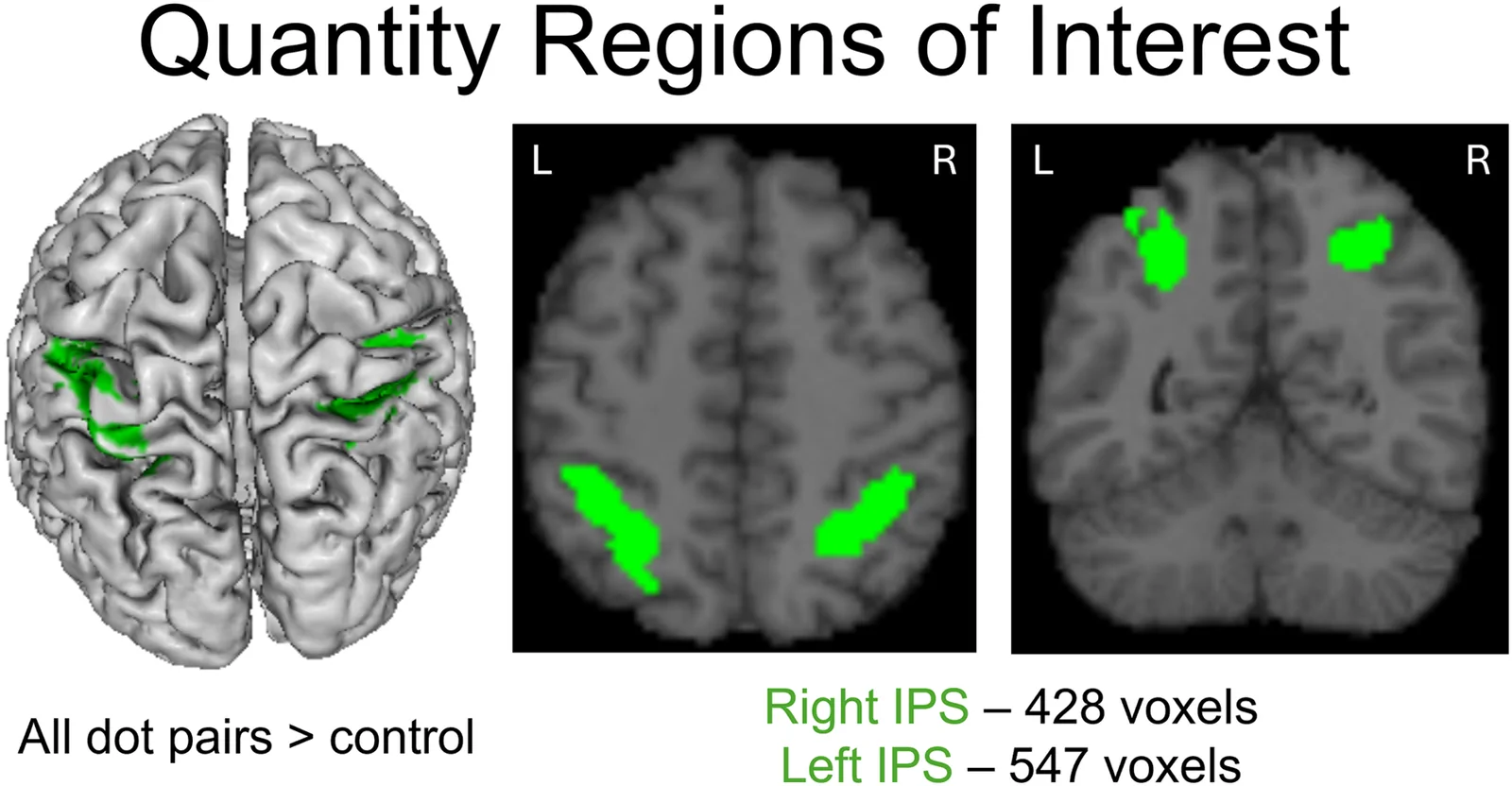
Regions of interest identified using the quantity localizer task. The voxels in green depict the 25% of voxels showing maximal activation (regardless of significance) for the contrast “all dot pairs vs. control” within the bilateral IPS.

Quantity combined ROIs were defined by constraining brain activation during the quantity localizer task (i.e., “all dot array pairs > control” contrast) within the left and right IPS. We then extracted the top 25% voxels showing maximal activation (regardless of significance) within the left and right hemispheres, which constituted our two quantity combined ROIs (see Figure 4). The resulting ROIs for right and left IPS were 428 voxels and 547 voxels, respectively.

#### Multiplication task analysis

For the multiplication task, first-level contrasts of “all problems > control”, “small problems > control”, and “large problems > control” were defined for each participant and each language. These contrasts were then included in a second-level analysis structured as a flexible factorial 2 × 2 repeated measures ANOVA. As noted above, activation was modeled as epochs with onset times time-locked to the presentation of the first spoken operand of the multiplication problem. Then, within each of the three ROIs (left STG/MTG, left IFG, and bilateral IPS), we compared brain activation elicited by the multiplication task in each language. For hypothesis 1, we compared the two languages collapsing across problem size using the contrasts “English [all problems – control] > Spanish [all problems – control]” or “Spanish [all problems – control] > English [all problems – control]”. For hypothesis 2, we compared the two problem sizes collapsing across languages using the contrasts “[Small – control] > [Large – control]” or “[Large – control] > [Small – control]”. Lastly, for hypothesis 3, we tested a Language (English, Spanish) by Problem Size (small, large) interaction within each of our defined ROIs, separately. This was done using the following contrasts: “English [small problems – large problems] > Spanish [small problems – large problems]” or “Spanish [large problems – small problems] > English [large problems – small problems]”.

Significance thresholds within the ROIs were determined using 3dClustSim (updated after December 2015; see https://afni.nimh.nih.gov/). This approach calculates the threshold for significant cluster sizes that would be unlikely to occur by chance within a masked brain volume at a specified uncorrected alpha level (for an example of how this approach has been applied to a parallel data set in balanced bilingual adults, see Cerda et al., 2024). Using 3dClustSim, we carried out 10,000 Monte Carlo simulations of random noise activations using a cluster-wise probability threshold of 0.05 and a voxel-wise threshold of 0.005 within each of our ROIs. The number of simulations in which clusters of different sizes appear within each ROI mask was tallied, and this data was used to calculate cluster size thresholds for significance. Additionally, we used 3dFWHMx to calculate the smoothness of the data for each participant, using a spatial autocorrelation function. These smoothness values were averaged across all participants and entered into 3dClustSim to calculate the cluster size needed for significance for each ROI. Clusters exceeding these size thresholds were considered significant. Based on this calculation, a cluster size of 47, 39, 15, and 12 voxels was needed for significance for the left STG/MTG, left IFG, left IPS, and right IPS ROIs, respectively (ACF values = 0.27, 5.79, 17.01).

As an exploratory analysis, we performed all the contrasts specified above at the whole brain level to determine if there were effects extending outside of our functional and anatomically defined ROIs. These whole brain analyses excluded all voxels within the ROIs to avoid multiple comparisons of the same data. At the whole brain level, a cluster size of 652 voxels was needed for significance based on the 3dClustSim calculation. To minimize potential bias, the initial whole brain analysis excluded predefined ROIs. To evaluate the robustness and completeness of the findings, we subsequently repeated the analysis including these ROIs.^1^ We added exploratory activation maps for the whole brain analysis with the ROIs included as confirmatory visualization in Figures 8 and 10.

## RESULTS

### Multiplication Task—Behavioral Results

First, we examined participants’ performance (accuracy and response time) on the multiplication task in English (L1) and Spanish (L2).^2^ For accuracy, a trial-level generalized linear mixed-effects model was created using the “lme4” package in R (Bates et al., 2015), with Subjects and Items (trials) as random effects. Because accuracy was a binary variable (correct vs. incorrect), we fit the generalized linear models with a logit link function. Fixed effects included Language (English, Spanish), Problem Size (Small, Large), Correctness (i.e., the correctness of the proposed solution: true, false), and Run (run 1, run 2). Correctness was included in this analysis as a factor of interest, given that the size of the Correctness effect (i.e., true vs. false problems) has been shown to be impacted by language in electrophysiological studies of bilingual arithmetic (Martinez-Lincoln et al., 2015; Salillas & Wicha, 2012). The model included a total of 4,608 within-subject observations from 32 subjects and 36 items per run. The model was fit using maximum likelihood with Laplace approximation.

For response time, a trial-level linear mixed model was created using the “lme4” package in R (Bates et al., 2015), including log-transformed RT with Subjects and Items (trials) as random effects. Fixed effects included Language, Problem Size, Correctness, and Run. Maximal models with a full random-effect structure were first computed. The maximal model did not converge, and we proceeded to reduce the complexity of the model incrementally, removing the least informative slope until the model converged. Satterthwaite’s method was used to estimate denominator degrees of freedom for F statistics. Only trials with accurate responses were included in response time analyses. Thus, the model included a total of 4,053 within-subject observations from 32 subjects.

#### Task accuracy

**Figure 5.**
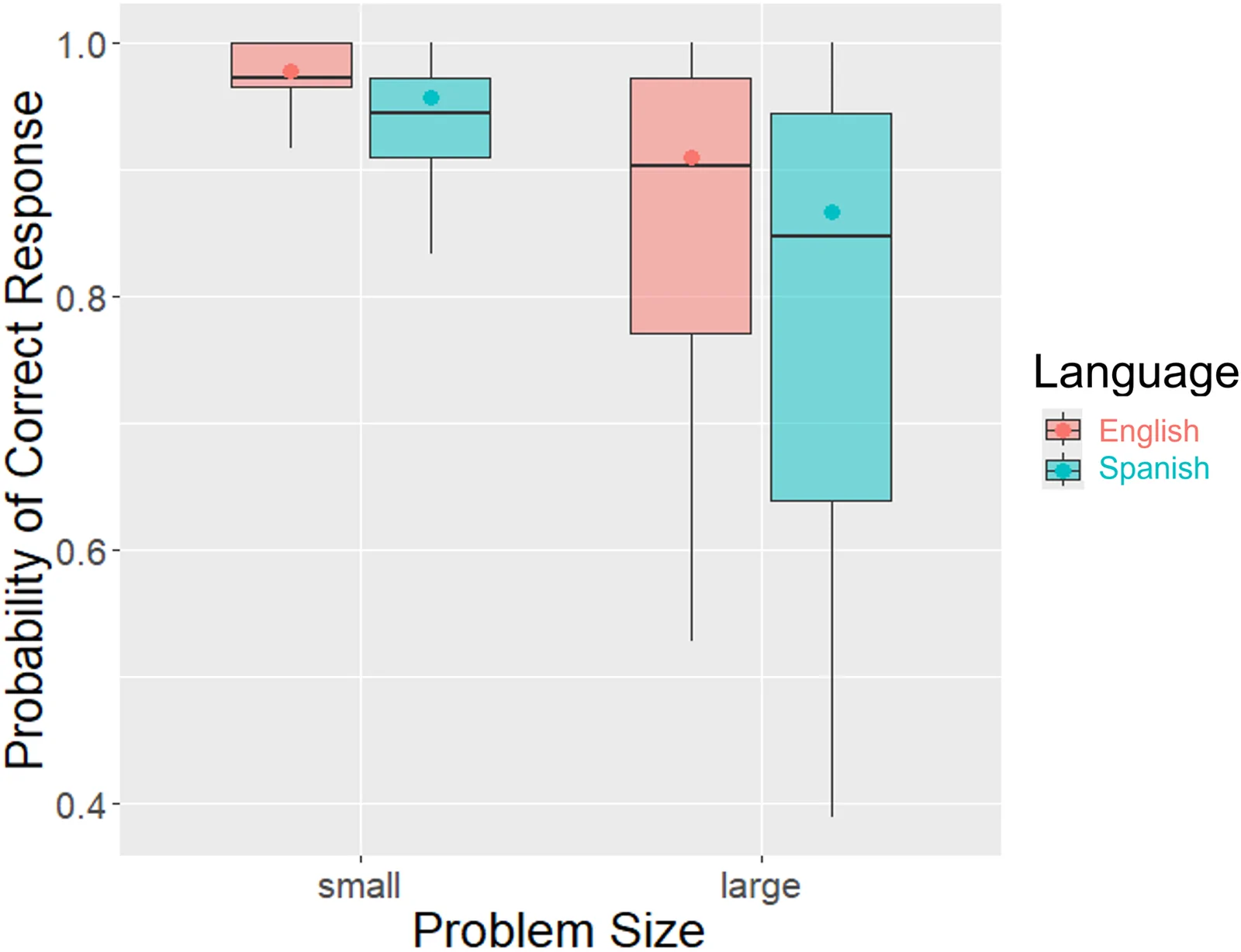
Multiplication task accuracy. A box and whisker plot depicting averaged subject-level data by condition, with small problems on the left, large problems on the right, separated by language. English is in red and Spanish is in blue. The size of the boxes represents the interquartile range, solid black bars within the boxes represent the median, and the whiskers represent the maximum and minimum values. The solid-colored dots represent the predicted probability of correct responses from the linear mixed model, where English is shown with the red dots and Spanish is shown in the blue dots. Note the significant main effects of Language and Problem Size, with no other significant interactions.

Accuracy was high in both languages with a main effect of Language (*β* = −0.66, *SE* = 0.24, *z* = −2.79, *p* < .01; odds ratio = 0.52; 95% CI [0.32, 0.82]), where participants were more accurate at responding to problems in English (average = 90.2%) than Spanish (average = 83.9%) (Figure 5A). As expected, there was a main effect of Problem Size (*β* = −1.45, *SE* = 0.26, *z* = −5.51, *p* < .001, odds ratio = 0.23, 95% CI [0.13, 0.39]) with higher accuracy for small (average = 94.0%) than large problems (average = 80.0%) (Figure 5B). Main effects of Correctness (odds ratio = 1.15, 95% CI [0.54, 2.45]) and Run (odds ratio = 1.15, 95% CI [0.95, 1.40]) were not significant (i.e., *p* > 0.05). Two-way interactions between Language and Problem Size (odds ratio = 1.25, 95% CI [0.72, 2.17]), Language and Correctness (odds ratio = 0.62, 95% CI [0.27, 1.39]), and Problem Size and Correctness (odds ratio = 0.53, 95% CI [0.44, 2.91]) were not significant (i.e., *p* > 0.05). Finally, the three-way interaction between these factors (odds ratio = 1.13, 95% CI [0.44, 2.91]) was also not significant (i.e., *p* > 0.05). Random intercept variance was larger for subjects (*SD* = 1.17) than for items (*SD* = 0.35), indicating greater variability across participants than across trials.

#### Task response time

**Figure 6.**
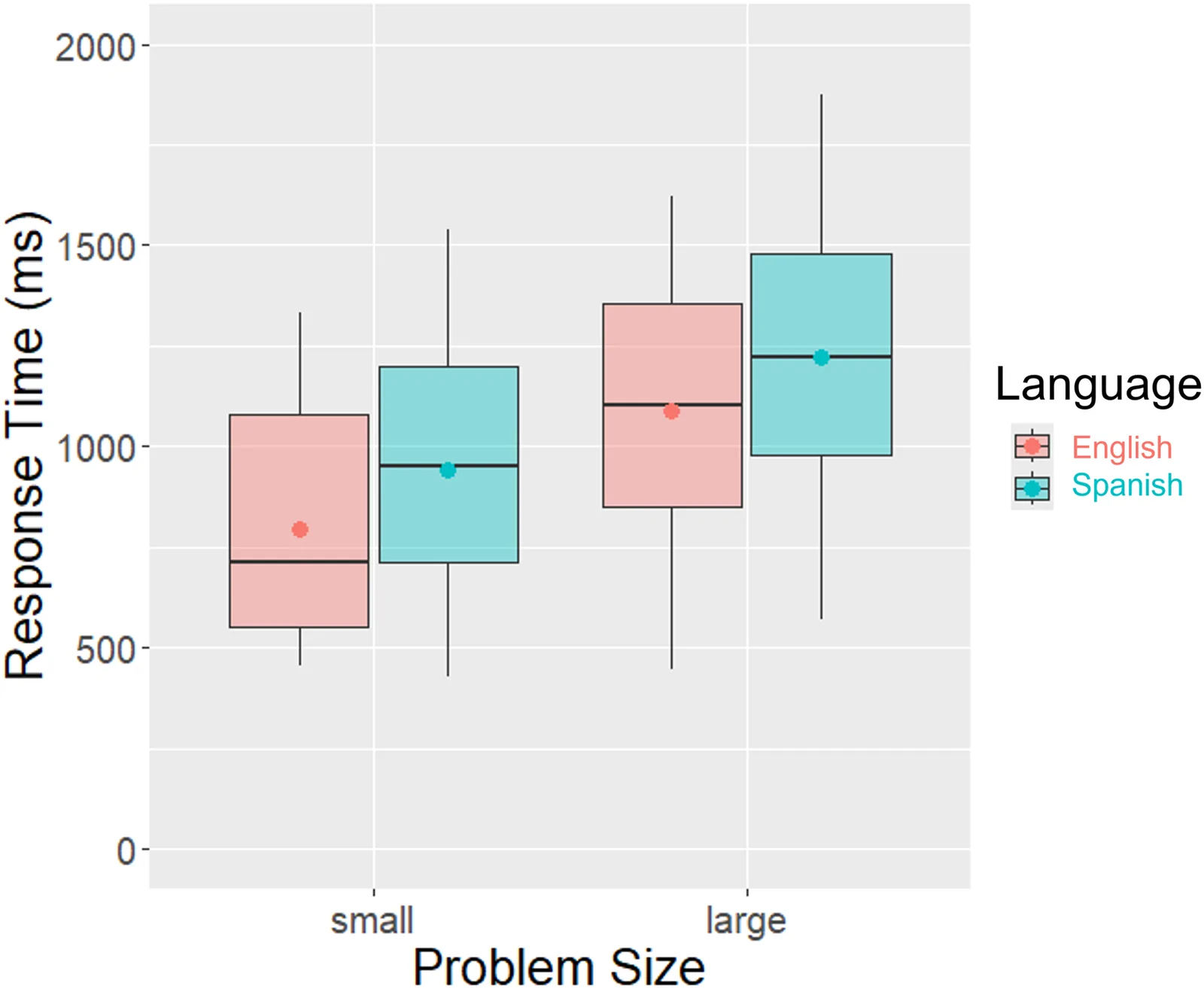
Multiplication task response times. A box and whisker plot depicting averaged subject-level data by condition, with small problems on the left, large problems on the right, separated by language. English is in red and Spanish is in blue. The size of the boxes represents the interquartile range, solid black bars within the boxes represent the median, and the whiskers represent the maximum and minimum values. The solid-colored dots represent the predicted response times from the linear mixed model, where English is shown with the red dots and Spanish is shown in the blue dots. Although the linear mixed model was constructed using log-transformed response time, all values have been reversed transformed to milliseconds for visualization purposes. Note the significant main effects of Language and Problem Size and the interaction between the two factors.

For Response Time, there was a main effect of Language (*β* = 0.17, *SE* = 0.03, 95% CI [0.11, 0.23], *F*(1, 33.2) = 26.7, *p* < 0.001) with slower responses to problems in Spanish (average = 1111 ms; *SE* = 46) than English (average = 988 ms; *SE* = 47) (Figure 6). There was a main effect of Problem Size (*β* = 0.29, *SE* = 0.03, 95% CI [0.23, 0.35], *F*(1, 49.2) = 110.8, *p* < 0.001) where responses were faster for small (average = 927 ms; *SE* = 44) than large problems (average = 1171 ms; *SE* = 49) (Figure 6B). There was also a main effect of Correctness (*β* = 0.19, *SE* = 0.03, 95% CI [0.12, 0.26], *F*(1, 47.2) = 61.7, *p* < 0.001), driven by faster responses to true (average = 965 ms; *SE* = 46) than false (average = 1133 ms; *SE* = 46) problems (Figure 6C). The main effect of Run was also significant (*β* = −0.02, *SE* = 0.01, 95% CI [−0.04, −0.005], *F*(1, 3907.1) = 6.36, *p* < 0.05), where participants were 2.6% faster in verifying problems in run 02 than run 01.

The interaction between Language and Problem Size was significant (*β* = −0.06, *SE* = 0.02, 95% CI [−0.11, −0.01], *F*(1, 3916.8) = 5.78, *p* < 0.05). Pairwise comparisons of estimated marginal means revealed that responses were faster in English than Spanish for both small problems (*β* = −0.171, *SE* = 0.03, *p* < 0.001) and large problems (*β* = −0.117, *SE* = 0.031, *p* < 0.01). A significant Problem Size effect was observed in both languages, with large problems producing slower responses than small problems in English (*β* = −0.315, *SE* = 0.030, *p* < .001) and Spanish (*β* = −0.261, *SE* = 0.030, *p* < .001). Thus, the interaction seemed to be driven by a larger Problem Size effect in English, where small problems were also significantly faster in English compared to Spanish.

Interactions between Language and Correctness (*β* = −0.01, *SE* = 0.03, 95% CI [−0.07, 0.04]), Problem Size and Correctness (*β* = 0.03, *SE* = 0.04, 95% CI [−0.04, 0.12]), and the three-way interaction between these factors (*β* = −0.01, *SE* = 0.03, 95% CI [−0.07, 0.10]) were not significant (i.e., *p* > 0.05). Random intercept variance was larger for subjects (*SD* = 0.32) than for items (*SD* = 0.04), indicating greater variability across participants than across trials.

#### Summary of behavioral results

As expected, participants verified the correctness of small multiplication problems faster and more accurately than large problems, which is consistent with the commonly reported Problem Size effect (see Zbrodoff & Logan, 2005, for review). A main effect of Problem Size was present, consistent with findings from early Spanish–English balanced bilingual adults on an identical task (Cerda et al., 2024). This sample of late Spanish learners were both faster and more accurate at responding to multiplication problems in English (L1) than Spanish (L2). Notably, accuracy was high in Spanish (mean 83.9%) despite having very low proficiency in Spanish. There was a significant interaction between Problem Size and Language for response time, driven by a larger Problem Size effect in English. No other interactions were significant for either measure.

### fMRI Results

#### Hypothesis 1: Main effect of language

**Figure 7.**
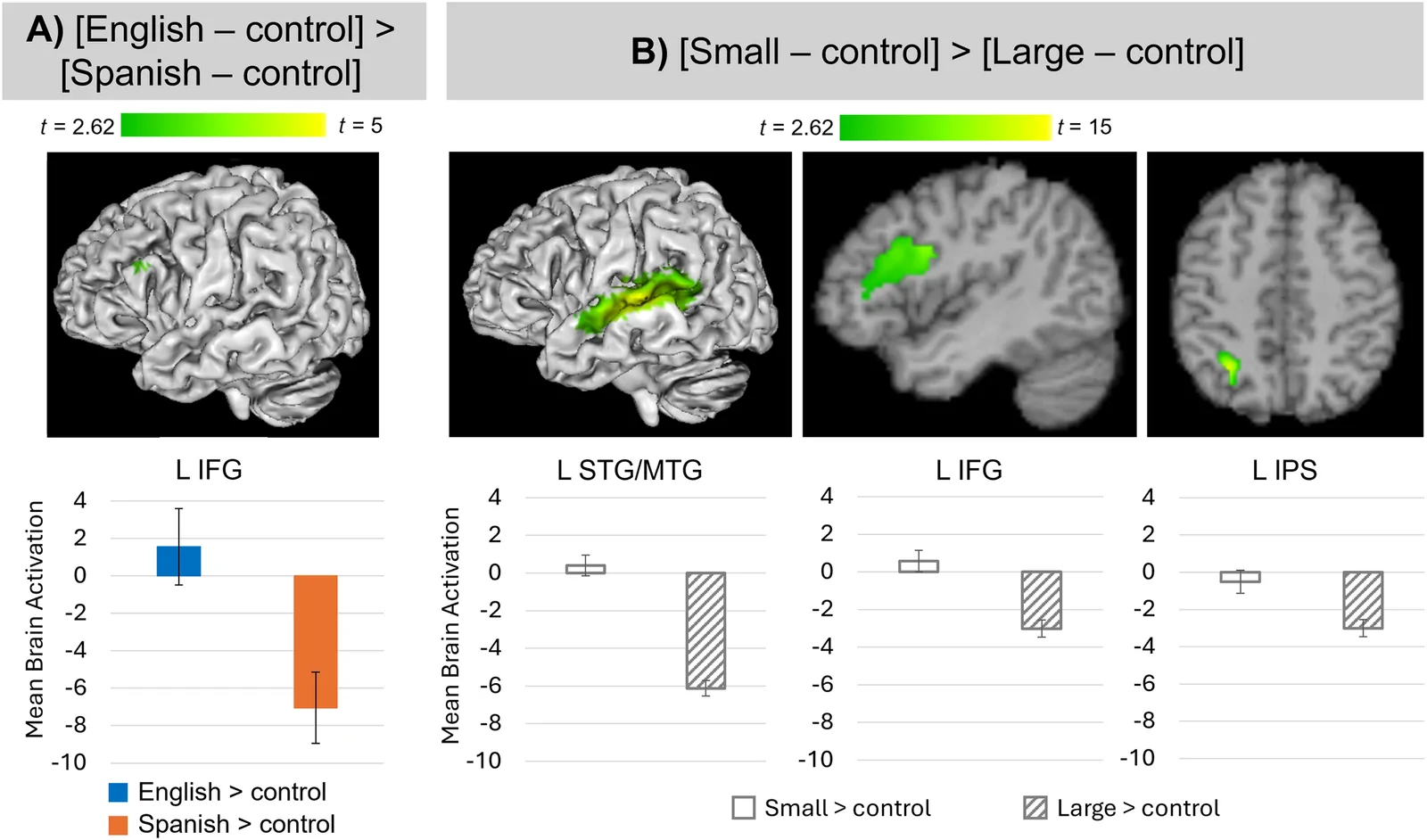
Significant main effects within the regions of interest. Activation maps within all regions of interest are depicted in a green color gradient. (A) Depiction of the brain activation for the contrast English [all problems – control] > Spanish [all problems – control] for the multiplication task on a rendered brain on the left hemisphere. A significant cluster for this contrast is shown in left IFG. Exploratory visualization of these clusters within the segments of the IFG revealed that all 60 voxels fell within the pars Triangularis with no voxels within the pars Opercularis or the pars Orbitalis. The bar graph directly below the rendered brain depicts the mean brain activation values within this cluster for English > control (blue) and Spanish > control (orange). (B) Depiction of the brain activation for the contrast [Small – control] > [Large – control] for the multiplication task, averaged across languages. Significant clusters for this contrast are shown in left STG/MTG (left panel), left IFG (middle panel), and left IPS (right panel). Exploratory visualization of significant clusters within the segments of the IFG revealed that 396 voxels fell within the pars Opercularis, 727 voxels fell within the pars Triangularis, and 32 voxels fell within the pars Orbitalis. Exploratory visualization of significant clusters within superior and middle temporal gyri revealed that 1396 voxels fell within the superior temporal gyrus and 669 voxels fell within the middle temporal gyrus. The bar graphs directly below each of the brain slices depict the corresponding mean brain activation values within these clusters for small > control (solid bars) and large > control (striped bars).

For the contrast “English [all problems – control] > Spanish [all problems – control]” we found significant clusters within the left IFG (see Figure 7A). When plotting the mean activation values for “English > control” and “Spanish > control” separately, this effect seemed to be driven by deactivation of this area in Spanish in comparison to English. More specific information about these clusters is shown in Table 1. No clusters reached significance within the left MTG/STG, bilateral IPS ROIs, or for the whole brain exploratory analysis. Additionally, no clusters reached significance for the reverse contrast: “Spanish [all problems – control] > English [all problems – control]”. To explore the relative deactivation for Spanish > control problems in the left IFG, we plotted the activation maps for each condition greater than control (i.e., English > control, Spanish > control) separately within the IFG and at the whole brain level (with ROI exclusion masks)^3^. However, this revealed no significant clusters for either contrast. Thus, there was no evidence that L2 shows activation in other parts of the IFG ROI that do not differ significantly from L1.

**Table 1.** Cluster information for the main effects for the multiplication task within regions of interest.

***Region of Interest***	**∼*BA***	**MNI Coordinates**			***k***	***z*-value**
		***X***	***Y***	***Z***		
**[English – control] > [Spanish – control]**						
Left IFG	44/45	−54	24	24	60	3.99
**[Spanish – control] > [English – control]**						
No significant clusters						
**[Small – control] > [Large – control]**						
Left STG/MTG	22/21	−60	−22	4	2,065	>8
Left IFG	44/45	−44	6	28	1,157	7.70
Left IPS	7	−30	−58	48	179	4.47
**[Large – control] > [Small – control]**						
No significant clusters						

*Note*. Cluster size (*k*), MNI coordinates of the peaks, *Z* values, and approximate Brodmann areas (∼BA) for the clusters showing significant activation for each contrast.

#### Hypothesis 2: Main effect of problem size

**Figure 8.**
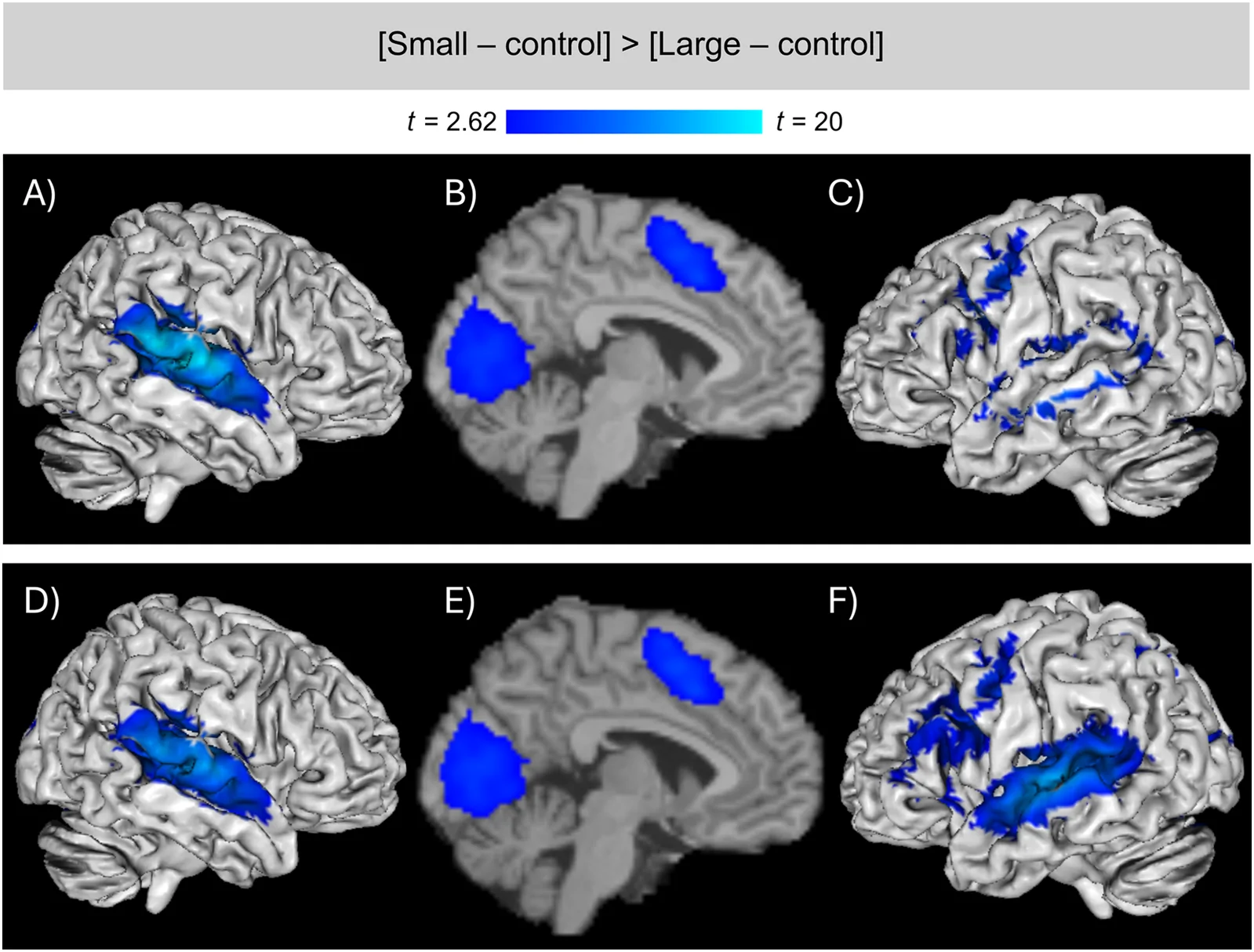
Main Effect of Problem Size at the whole brain level. The top row depicts activation maps for the contrast Small [all problems – control] > Large [all problems – control] for the multiplication task outside of regions of interest (i.e., using exclusion masks of our regions of interest in bilaterial IPS, left STG/MTG, and left IFG) in a blue color gradient. The bottom row depicts activation maps for the contrast Small [all problems – control] > Large [all problems – control] for the multiplication task with no exclusion masks for the regions of interest in a blue color gradient. (A and D) Show significant clusters on rendered brains on the right hemisphere. (B and E) Show significant clusters on medial sagittal slices of the brain. (C and F) Show significant clusters on rendered brains on the left hemisphere. Note that these clusters were outside of our combined functional-anatomical ROIs.

The analysis carried out to investigate the main effect of Problem Size included multiplication problems averaged across languages. For the contrast “[Small – control] > [Large – control]”, we found significant clusters within the left STG/MTG, left IFG, and left IPS (see Figure 7B). When plotting the mean activation values for “Small > control” and “Large > control” separately, these effects seemed to be driven by deactivation of all three regions for large problems (see Figure 7B). More specific information about these clusters is shown in Table 1. No clusters reached significance within the right IPS. Additionally, no clusters reached significance within these ROIs for the reverse contrast “[Large – control] > [Small – control]”. In an exploratory analysis of the whole brain, there were significant clusters for the contrast “[Small – control] > [Large – control]” (See Table 2 and Figure 8). No clusters reached significance for the reverse contrast, “[Large – control] > [Small – control]”. Similar to hypothesis 1, an analysis was added to explore the relative deactivation for large > control problems in the ROIs, we plotted the activation maps for each condition greater than control (i.e., small > control, large > control) separately within the ROIs and at the whole brain level (with ROI exclusion masks). However, this revealed no significant clusters for either contrast.

**Table 2.** Cluster information for the main effects for the multiplication task at the whole brain level.

***Anatomical location***	**∼*BA***	**MNI Coordinates**			***k***	***z*-value**
		***X***	***Y***	***Z***		
**[English – control] > [Spanish – control]**						
No significant clusters						
**[Spanish – control] > [English – control]**						
No significant clusters						
**[Small – control] > [Large – control]**						
Right Superior Temporal Gyrus	22	56	−14	4	4,055	>8
Left Superior Temporal Gyrus*	22	−40	−36	14	1,961	>8
Left Inferior Frontal Gyrus* & Precentral Gyrus	44/45	−44	6	30	3,941	7.79
Left Cuneus	17	−8	−78	12	7,874	6.36
**[Large – control] > [Small – control]**						
No significant clusters						

*Note*. Cluster size (*k*), MNI coordinates of the peaks, *Z* values, and approximate Brodmann areas (∼BA) for the clusters showing significant activation for each contrast in the whole brain analysis using exclusion masks for the regions of interest.

* Note that these clusters were outside of our combined functional-anatomical ROIs for left STG/MTG and left IFG.

#### Hypothesis 3: Language by problem size interaction

The analysis testing for an interaction between Language (English, Spanish) and Problem Size (small, large) resulted in no significant clusters within the regions of interest, or in whole brain analysis.

#### Planned exploratory analyses: Second language proficiency and the language bias for arithmetic

Given that proficiency in each language can determine whether there is a difference in performance on arithmetic tasks across languages (e.g., Garcia et al., 2021), two additional analyses were performed to explore whether language proficiency modulates performance and brain activation patterns during the multiplication task. We predicted that L2 learners with lower proficiency in Spanish might show increased differences between languages on the multiplication task than learners with higher Spanish proficiency.

We measured multiple dimensions of language proficiency (i.e., picture vocabulary, oral comprehension, self-reported proficiency). Based on prior work, we chose to use one standardized measure of language proficiency in these analyses, picture vocabulary, as a proxy of language skill. Previous bilingual language studies used the Boston Naming Test as a proxy for bilingual language proficiency to investigate processing differences across languages in the brain (Hernandez et al., 2000; Moreno & Kutas, 2005; Salillas & Wicha, 2012). The Boston Naming Test requires participants to orally name a series of line drawings in each language. Herein, the Picture Vocabulary subtest from the Woodcock-Johnson Tests of Achievement (English) and the equivalent Vocabulario Sobre Dibujos subtest of the Batería III (Spanish; see Standardized Measures section above) similarly measure language proficiency through picture naming and identification.

First, we studied the effect of Spanish picture naming proficiency on response time and accuracy on the multiplication task. We predicted a negative relation between Spanish proficiency and language-related differences in arithmetic performance, where lower Spanish proficiency would result in larger performance differences across languages. More specifically, for our L2 learners, we expected lower Spanish proficiency to be associated with poorer Spanish performance on the multiplication task, particularly for large problems.

We conducted separate hierarchical regression analyses for accuracy and response times. English (i.e., L1) vocabulary proficiency was entered into the models as a covariate of no interest and Spanish vocabulary proficiency as the independent variable. The rationale for including English proficiency as a covariate of no interest was to account for between-subject variability in general language skill. The dependent measure was the difference in task performance in multiplication across languages (i.e., English accuracy – Spanish accuracy and English response time – Spanish response time). These analyses revealed no significant relationship between the language differences in arithmetic and Spanish vocabulary proficiency, for either accuracy or response times, above and beyond English vocabulary proficiency (see Table 3).

**Table 3.** Hierarchical models exploring the relation between Spanish (L2) proficiency and language differences in arithmetic.

**Model**	**Predictors**	**Small Problems**		**Large Problems**	
		***R*^2^Δ**	***p*-value**	***R*^2^Δ**	***p*-value**
**Accuracy (English–Spanish)**					
1	English Proficiency	0.3804	<0.001	0.00309	0.76
2	English Proficiency	0.0028	0.72	0.0003	0.92
	Spanish Proficiency				
	**Overall Model**		<0.001		0.95
**Response Time (English–Spanish)**					
1	English Proficiency	0.06846	0.14	0.0023	0.79
2	English Proficiency	<0.0001	0.99	0.0016	0.82
	Spanish Proficiency				
	**Overall Model**		0.35		0.94

*Note*. For both Accuracy and Response Time (RT), language differences for performance on small problems and large problems were modeled separately. The additional variance explained (*R*^2^Δ) and corresponding *p*-values are shown.

Second, we completed a similar analysis using brain activation for the contrast [Spanish – control] > [English – control] for small and large problems, separately, within each region of interest (left STG/MTG, left IFG, and bilateral IPS). Overall, we expected that lower Spanish proficiency would be associated with larger language-related differences within each ROI. Because greater frontal-parietal activation has been associated with more effortful processing or reliance on quantity processes, we predicted a negative relation between Spanish proficiency and activation in both left IFG and bilateral IPS for the contrast [Spanish – control] > [English – control]. We predicted that this effect would be driven by a negative relation between Spanish proficiency and activation for the Spanish multiplication task (i.e., Spanish > control), and this relation would be significant for both small and large problems, although stronger for larger problems (Prado et al., 2011, 2014). We also predicted that a positive relation between Spanish proficiency and activation in STG/MTG might be revealed for the contrast [Spanish – control] > [English – control]. This effect was expected to be driven by a positive relation between Spanish proficiency and activation for the Spanish multiplication task (i.e., Spanish > control). We predicted that these relations would be significant for both small and large problems (vs. control), although stronger for small problems (Prado et al., 2011, 2014).

To address these hypotheses, a hierarchical regression analysis was performed with English vocabulary proficiency entered into the model as a covariate of no interest and Spanish vocabulary proficiency as the independent variable. The dependent measure was brain activation for the contrast [Spanish – control] > [English – control] for small problems and large problems, separately, within each ROI. These analyses revealed no significant relationships between Spanish > English brain activation and Spanish vocabulary proficiency above and beyond English vocabulary proficiency (see Table 4) in any of the ROIs.

**Table 4.** Hierarchical models exploring the relation between Spanish proficiency and language differences in brain measures taken from our regions of interest, left STG/MTG, left IFG, and bilateral IPS.

**Model**	**Predictors**	**Small Problems**		**Large Problems**	
		***R*^2^Δ**	***p*-value**	***R*^2^Δ**	***p*-value**
**Left STG/MTG**					
1	English Proficiency	0.00295	0.76	0.0047	0.70
2	English Proficiency	0.00428	0.72	0.0001	0.95
	Spanish Proficiency				
	**Overall model**		0.90		0.93
**Left IFG**					
1	English Proficiency	0.0003	0.92	0.0300	0.34
2	English Proficiency	0.01645	0.49	0.0183	0.46
	Spanish Proficiency				
	**Overall model**		0.78		0.48
**Left IPS**					
1	English Proficiency	0.00856	0.61	0.0037	0.73
2	English Proficiency	<0.0001	0.99	0.0205	0.44
	Spanish Proficiency				
	**Overall model**		0.88		0.69
**Right IPS**					
1	English Proficiency	0.01783	0.46	0.00576	0.67
2	English Proficiency	0.0002	0.93	0.0043	0.72
	Spanish Proficiency				
	**Overall model**		0.76		0.86

*Note*. For all regions of interest, language differences for small problems and large problems were modeled separately. The additional variance explained (*R*^2^Δ) and corresponding *p*-values are shown.

#### Exploratory analyses of only true problems

In our analysis of task performance, we found main effects of Language and Problem Size for both response time and accuracy. These results were obtained from updated behavioral analyses that account for trial-level performance, per reviewer request. In a previous analysis, we had originally found evidence of an interaction between Problem Size and Correctness for accuracy, driven by higher accuracy when verifying true solutions than false solutions for large problems, but not for small problems. This interaction was no longer significant in the updated analysis. Based on the original findings, we explored the possibility that the Problem Size effect patterns in the measures of brain activity might be influenced by the inclusion of false solutions that are more difficult to process and may involve error processing mechanisms. To address that possibility, we repeated our analyses of behavioral and brain measures including only multiplication problems with correct proposed solutions (i.e., true problems). Although there was no longer evidence for an interaction between Problem Size and Correctness in behavioral measures, for transparency, we report the results of the analysis below. This analysis also parallels analyses that were completed in Cerda et al. (2024) from balanced bilinguals completing an identical task.

The analysis of only true problems did not change the pattern of behavioral results. For accuracy, both the main effects of Language (*β* = −0.66, *SE* = 0.23, *z* = −2.8, *p* < 0.01; odds ratio = 0.51; 95% CI [0.32, 0.81]) and Problem Size (*β* = −1.48, *SE* = 0.28, *z* = −5.15, *p* < .001, odds ratio = 0.22, 95% CI [0.12, 0.39]) remained significant, and no interactions were significant (*p* > 0.05). For response time, both main effects of Language (*β* = 0.17, *SE* = 0.03, 95% CI [0.11, 0.24], *F*(1, 31.8) = 22.8, *p* < 0.001) and Problem Size (*β* = 0.29, *SE* = 0.012, 95% CI [0.22, 0.36], *F*(1, 40.4) = 58.0, *p* < 0.001) and the interaction between the two factors remained significant (*β* = −0.06, *SE* = 0.02, 95% CI [−0.11, −0.01], *F*(1, 2643.6) = 6.07, *p* < 0.05). Pairwise comparisons of estimated marginal means revealed that responses were faster in English than Spanish for both small problems (*β* = −0.176, *SE* = 0.03, *p* < 0.001) and large problems (*β* = −0.112, *SE* = 0.031, *p* < 0.01). A significant Problem Size effect was observed in both languages, with large problems producing slower responses than small problems in English (*β* = −0.297, *SE* = 0.037, *p* < .001) and Spanish (*β* = −0.233, *SE* = 0.037, *p* < .001). Thus, the interaction still seemed to be driven by a larger Problem Size effect in English, where small problems were also significantly faster in English compared to Spanish. Hierarchical regression analyses revealed no significant relations between the language effect and Spanish vocabulary proficiency, for either accuracy or response time, above and beyond English vocabulary proficiency, consistent with our previous analysis that included both true and false solutions.

**Figure 9.**
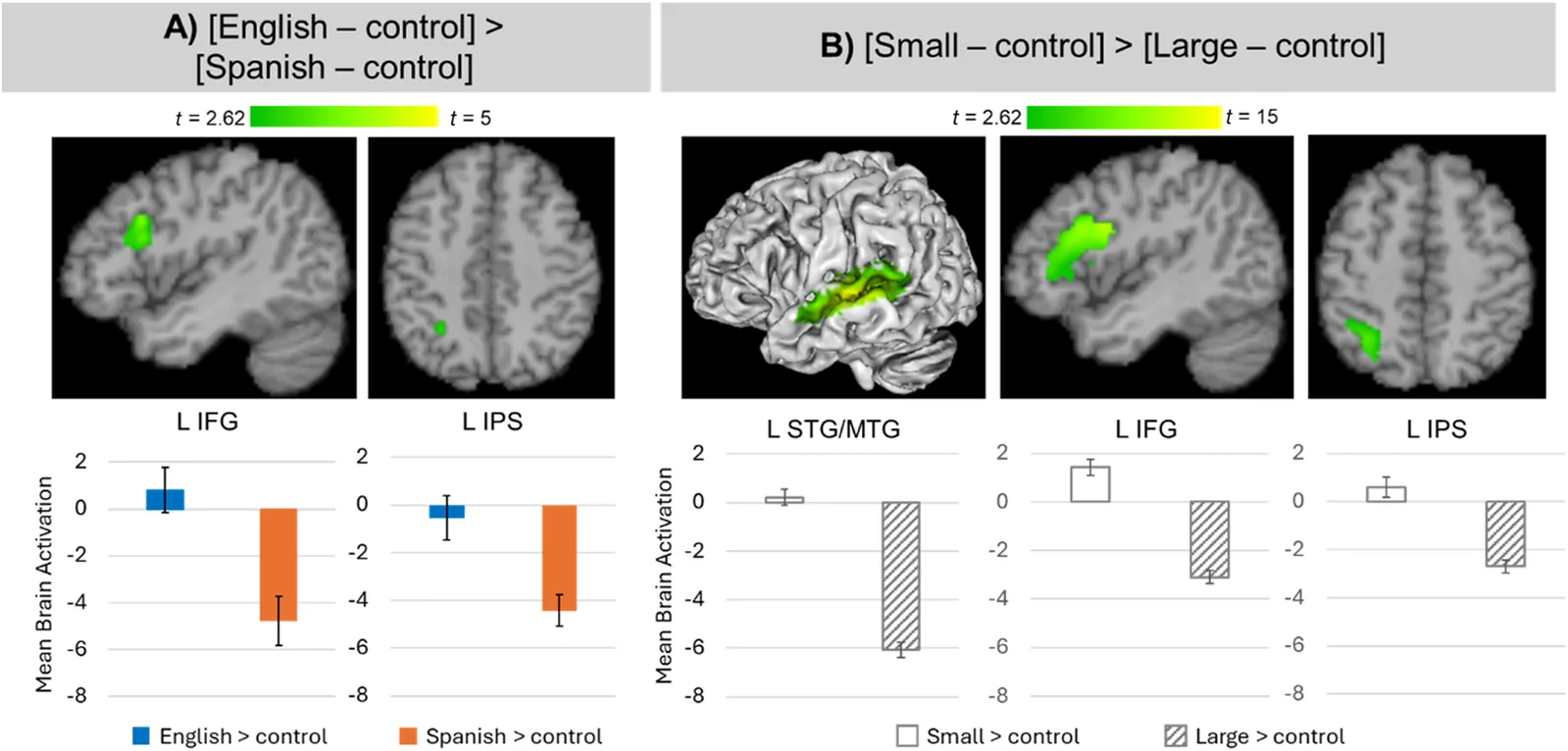
Analysis of true problems: main effects within the regions of interest. Activation maps within all regions of interest are depicted in a green color gradient. (A) Depiction of the brain activation for the contrast [English – control] > [Spanish – control] for the multiplication task. Significant clusters are shown in left IFG (left) and the left IPS (right). The bar graphs below the brain slices depict the corresponding mean brain activation values within both clusters for English > control (blue) and Spanish > control (orange). (B) Depiction of the brain activation for the contrast [Small – control] > [Large – control] for the multiplication task collapsed across languages. Significant clusters are shown in left STG/MTG (left panel), left IFG (middle panel), and left IPS (right panel). The bar graphs below the brain slices depict the corresponding mean brain activation values within all clusters for small > control (solid bars) and large > control (striped bars).

Exclusion of false solutions did affect the pattern of effects in the measures of brain activity. A main effect of Language, with significant clusters for the contrast [English – control] > [Spanish – control], was observed again for the left IFG, as well as a new effect for the left IPS (see Figure 9A). For both regions, the effect was due to deactivations for Spanish. The main effect of Problem Size was consistent with our previous analysis, with significant clusters for the contrast [Small – control] > [Large – control] in the left STG/MTG, left IFG, and left IPS (Figure 9B). The effects were again due to deactivation for large problems. Information about these clusters can be found in Table 5.

**Table 5.** Cluster information of the main effects for the multiplication task for only true problems within regions of interest.

***Region of Interest***	**∼*BA***	**MNI Coordinates**			***k***	***z*-value**
		***X***	***Y***	***Z***		
**[English – control] > [Spanish – control]**						
Left IFG	44/45	−50	22	24	256	3.99
Left IPS	7	−28	−54	46	42	2.96
**[Spanish – control] > [English – control]**						
No significant clusters						
**[Small – control] > [Large – control]**						
Left STG/MTG	22/21	−60	−22	4	2,060	>8
Left IFG	44/45	−42	6	28	1,400	>8
Left IPS	7	−30	−56	46	366	7.12
**[Large – control] > [Small – control]**						
No significant clusters						

*Note*. Cluster size (*k*), MNI coordinates of the peaks, *Z* values, and approximate Brodmann areas (∼BA) for the clusters showing significant activation for each contrast.

**Figure 10.**
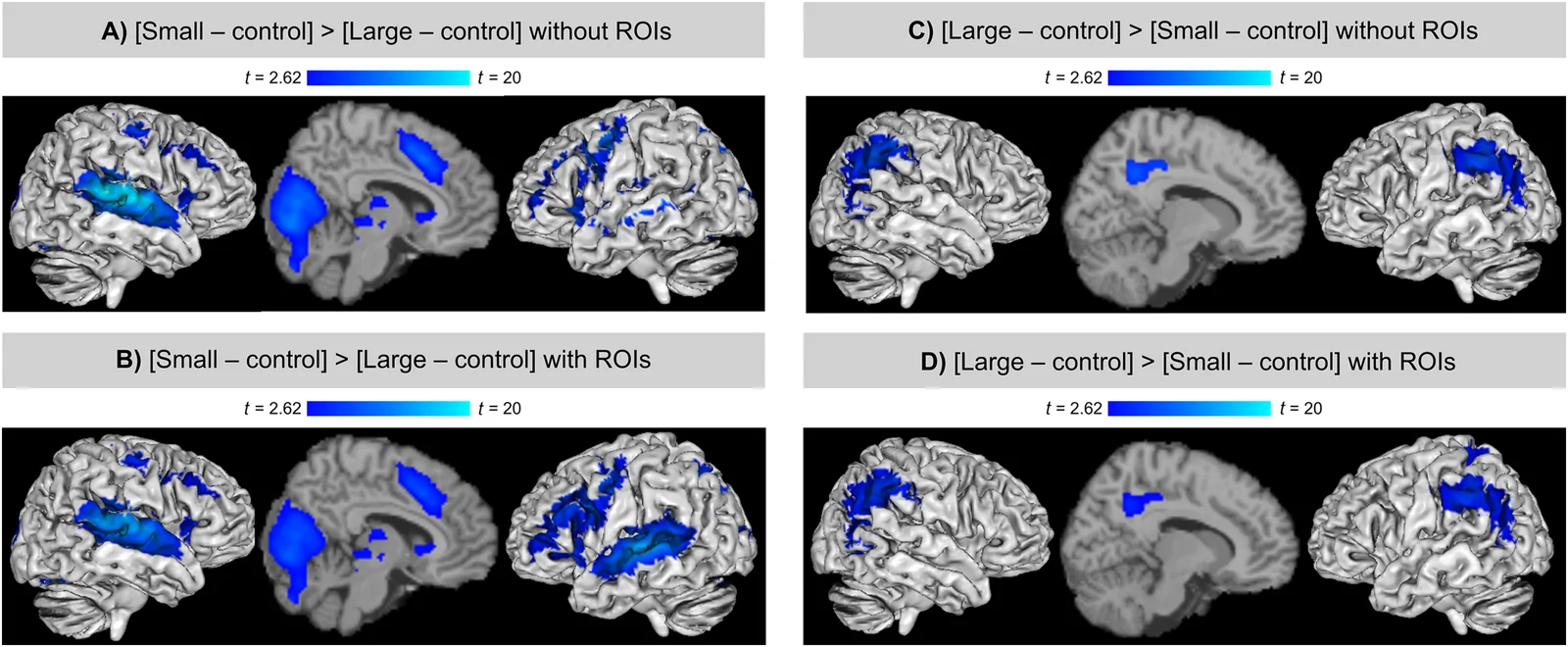
Analysis of true problems: main effect of Problem Size at the whole brain level. Activation maps outside of the regions of interest are depicted in a blue color gradient. Depiction of the brain activation for the contrast Small [all problems – control] > Large [all problems – control] for the multiplication task without the ROIs (A) and with the ROIs included (B). Depiction of the brain activation for the contrast Large [all problems – control] > Small [all problems – control] for the multiplication task without the ROIs (C) and with the ROIs included (D).

To determine if there were effects extending outside of our ROIs, we conducted an exploratory analysis of the whole brain for all contrasts. We found significant clusters for the contrasts [Small – control] > [Large – control] and [Large – control] > [Small – control] (see Figure 10). Table 6 gives specific information about these significant clusters. We added exploratory activation maps for the whole brain analysis with the ROIs included as confirmatory visualization in Figure 10^4^. Hierarchical regression analyses revealed no significant relationship between language differences in brain measures within our ROIs and Spanish vocabulary proficiency above and beyond English vocabulary proficiency, consistent with our previous analyses.

**Table 6.** Cluster information for the main effects for the multiplication task for only true problems at the whole brain level.

***Anatomical location***	**∼*BA***	**MNI Coordinates**			***k***	***z*-value**
		***X***	***Y***	***Z***		
**[English – control] > [Spanish – control]**						
No significant clusters						
**[Spanish – control] > [English – control]**						
No significant clusters						
**[Small – control] > [Large – control]**						
Right Superior Temporal Gyrus	22	54	−16	4	3,720	>8
Left Inferior Frontal Gyrus* & Precentral Gyrus	44/45	−42	6	30	7,717	>8
Left Superior Temporal Gyrus	22	−42	−32	8	1,627	>8
Left Lingual Gyrus	18	−8	−78	10	12,078	>8
Right Claustrum	NA	32	26	0	1,134	6.54
Right Precentral Gyrus	4	54	−4	44	1,550	5.93
Left Thalamus/Pulvinar	NA	−4	−32	−4	956	4.44
**[Large – control] > [Small – control]**						
Right Supramarginal Gyrus	40	60	−44	34	2,269	6.69
Left Inferior Parietal Lobule	40	−62	−38	34	1,696	6.50
Right Precuneus	31	12	−50	38	818	4.91

*Note*. Cluster size (*k*), MNI coordinates of the peaks, *Z* values, and approximate Brodmann areas (∼BA) for the clusters showing significant activation for each contrast using exclusion masks for the regions of interest.

* Note that these clusters were outside of our combined functional-anatomical ROIs.

#### Exploratory analysis of language without control contrast

**Figure 11.**
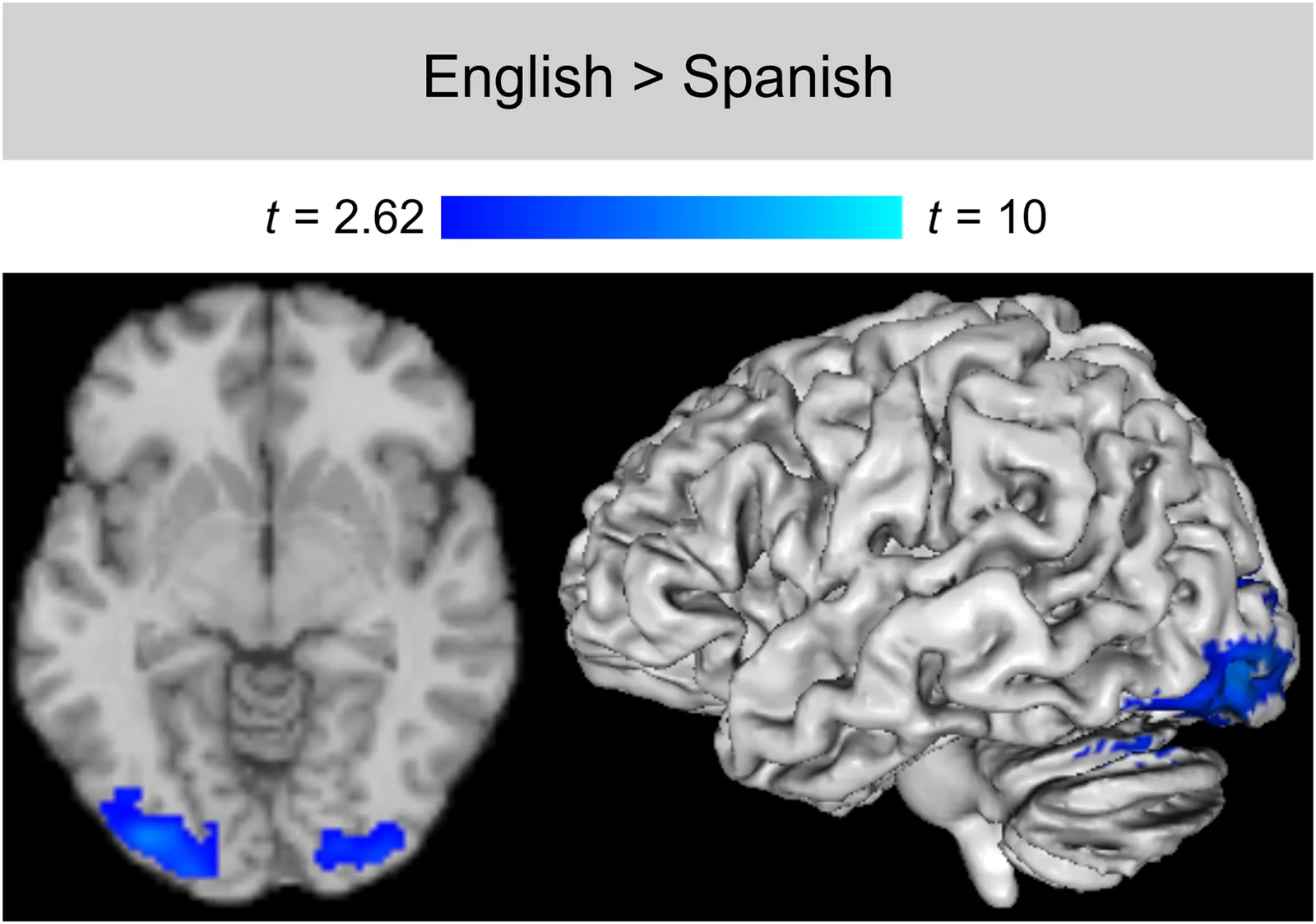
Main effect of Language without the control condition. Activation maps outside of the regions of interest are depicted in a blue color gradient. Depiction of the brain activation for the contrast English > Spanish for the multiplication task on a horizontal slice (left) and on a rendered brain on the left hemisphere (right).

We completed an exploratory analysis of problems in English > Spanish and Spanish > English without the control condition within each of the ROIs and at the whole brain level (with ROI exclusion masks)^5^. This new analysis revealed no significant activation within our regions of interest (i.e., left STG/MTG, left IFG, and bilateral IPS) for the English > Spanish or Spanish > English contrast. However, we did find significant activation at the whole brain level in bilateral occipital cortex for the contrast English > Spanish (Figure 11). Information about these clusters can be found in Table 7.

**Table 7.** Cluster information for the main effect of Language for the multiplication task without the control condition at the whole brain level.

***Anatomical location***	**∼*BA***	**MNI Coordinates**			***k***	***z*-value**
		***X***	***Y***	***Z***		
**English > Spanish**						
Left Middle Occipital Gyrus	18	−32	−86	−8	1,388	5.59
Right Inferior Occipital	18	36	−92	−2	711	4.67
**Spanish > English**						
No significant clusters						

*Note*. Cluster size (*k*), MNI coordinates of the peaks, *Z* values, and approximate Brodmann areas (∼BA) for the clusters showing significant activation for each contrast using exclusion masks for the regions of interest.

## DISCUSSION

The goal of this study was to determine whether adult learners of an L2 with limited L2 proficiency engage shared or separate cortical regions to process simple arithmetic in their L1 and L2. In brief, adult learners of Spanish as an L2 verified simple multiplication problems faster and more accurately in their L1 (English) than L2 (Spanish). Imaging data revealed that these adults differentially engaged the left IFG across languages when verifying multiplication problems, driven by less activation for L2. Additionally, they showed less engagement of the left STG/MTG, the left interparietal sulcus (IPS), and the left IFG for large problems (e.g., 9 × 8 = 72) than small problems (e.g., 2 × 4 = 8). We discuss the implications of these results below.

### Second Language Learners Show Less Engagement of Effortful, Arithmetic Retrieval Processes in Their L2

Adult Spanish learners were faster and more accurate at verifying the correctness of multiplication problems presented as auditory number words in their L1 (English) compared to later-learned L2 (Spanish). We also found an interaction between Language and Problem Size, discussed below. It was expected that they would perform better in their L1 as it is their strongly dominant language and the language used for learning and rehearsing simple arithmetic facts in childhood. These findings are consistent with previous reports of a language bias for arithmetic processing (Cerda et al., 2019, 2023; Dehaene et al., 1999; Frenck-Mestre & Vaid, 1993; Lotus Lin et al., 2019; Marsh & Maki, 1976; Salillas & Wicha, 2012; Spelke & Tsivkin, 2001; Tamamaki, 1993; Van Rinsveld et al., 2016, 2017).

Previous work suggests that bilinguals with lower L2 proficiency elicit larger differences in performance across languages on arithmetic and numeric tasks than individuals with higher L2 proficiency (Garcia et al., 2021). We did not find evidence of a significant relationship between language differences for arithmetic performance (i.e., accuracy or response times) and relative Spanish vocabulary proficiency within our sample. This is likely because our L2 learner population showed limited variability in Spanish vocabulary proficiency. Indeed, all participants scored within the “very low” range on this measure. However, in the context of the bilingual arithmetic literature, our behavioral results add to the existing evidence suggesting that language differences in arithmetic are more pronounced for populations with lower L2 proficiency in comparison to populations with higher L2 proficiency. Previous research with early, balanced bilingual adults performing this same multiplication verification task showed more inconsistent and subtler differences across languages, usually under higher task demands, such as faster response times in the language of learning partially attributable to increased demands of hearing arithmetic problems in an MRI scanner (Cerda et al., 2024) or brain activity differences for harder problems only under time pressure (Cerda et al., 2023).

Our imaging data, although unexpected, may provide insight into the potential driver of these behavioral differences across languages. Contrary to the predictions that we made based on the literature, we observed no differences in STG/MTG, which might have suggested differential engagement of memory retrieval. Instead, we observed an effect of Language on cortical activation patterns in the IFG and IPS, but in the opposite direction than anticipated, with *less* activation for L2 than L1. In an exploratory analysis at the whole brain level, we also found that L1 engaged areas of bilateral occipital cortex to a greater extent than L2. To contextualize these findings, we discuss what is known about the engagement of these regions in arithmetic and bilingualism.

Leading math cognition models suggest that differences in performance across languages should stem primarily from differences in direct retrieval of the arithmetic facts (Campbell, 1994; Campbell & Clark, 1992; Campbell & Epp, 2004; Campbell et al., 1999; Dehaene et al., 1999; Spelke & Tsivkin, 2001). The left STG/MTG are thought to be involved in the storage of arithmetic facts in verbal memory, especially when the facts have been explicitly memorized (Prado et al., 2013). One study (Van Rinsveld et al., 2017) observed that temporal regions, including the superior temporal gyrus, were activated to a greater extent in German (L1 and the language of learning) when bilingual adults processed simple addition compared to French (L2). This was interpreted as reflecting less reliance on direct semantic associations between problems and solutions in the weaker language.

Given this context, we hypothesized that adult L2 learners would engage STG/MTG to a greater degree when verifying arithmetic in L1 (English) than L2 (Spanish). However, we found no significant differences in the STG/MTG when directly contrasting both languages. Notably, accuracy was high in L2 (83.9%), suggesting that these adults were able to verify simple multiplication facts in L2 even with limited L2 proficiency, although slower and less accurately than in L1. In turn, our observed language effects on accuracy and response time were not driven by differential engagement of the verbal representations for multiplication facts across languages. Instead, the language differences we observed in IFG and IPS suggest that the source of the behavioral effects may be differential engagement of fronto-parietal cortical regions, although perhaps not in the expected direction.

Engagement of a fronto-parietal network has been observed for more effortful arithmetic processes (De Smedt et al., 2011). The left IFG is often thought to be involved in the effortful control and retrieval of semantic knowledge. Specifically, the left IFG seems to be critical for selecting between active representations in memory, especially during effortful retrieval of less practiced arithmetic facts, such as large multiplication problems (Prado et al., 2011). Additionally, activation of IPS is thought to reflect involvement of quantity-based processes, such as calculation (De Smedt et al., 2011; Prado et al., 2013). In bilingual arithmetic studies, greater engagement of IFG has been observed in L2 than L1, and has been interpreted as reflecting translation of arithmetic problems from L2 to L1 (Venkatraman et al., 2006; Wang et al., 2007). For example, Wang et al. (2007) asked native Chinese speakers who were late learners of English to verify multiplication problems that explicitly required calculations (e.g., “18 times 2 is 46?”). They found that the left IFG was activated only for L2, and took this to suggest that L2 learners engaged additional effort to translate the problem from L2 to L1 to retrieve the answer in L1. Similarly, Venkatraman et al. (2006) trained English–Chinese bilinguals on novel base-7 addition problems only in one language and found that the left IFG was engaged to a greater extent for the untrained language compared to the trained language. They took this to suggest that the untrained language required greater effort to translate the problem to the trained language to retrieve the answer by rote.

Based on these findings, we predicted that L2 would engage these fronto-parietal regions (i.e., the IFG and IPS) to a greater degree than L1, reflecting more effortful or additional processing in the weaker language. Indeed, our robust behavioral differences with slower and less accurate responses in L2 suggest more effortful processing in L2 than L1. Yet, we measured less engagement of the left IFG for problems in L2 than L1 (Figure 7A), as well as less engagement of IPS for true problems in L2 than L1 (Figure 9A). The observed deactivation of IFG for L2 problems may reflect the general inability of L2 learners to engage effortful retrieval processes for arithmetic presented in their weaker language, L2. As mentioned, left IFG has been associated with processes related to effortful retrieval of arithmetic facts (Prado et al., 2011). Moreover, activity in the left IFG decreases as young children master multiplication facts, suggesting less reliance on numerical processes to favor more efficient retrieval (Prado et al., 2014). These findings would suggest that the IFG is more engaged when arithmetic is more effortful. However, these studies reflect typical development of arithmetic processing in monolinguals when simple multiplication problems are presented as Arabic digits (e.g., 2 × 4 = 8). The current study imposes additional demands on our participants, where they must process simple arithmetic problems presented as auditory number words in a less proficient L2. The deactivation of IFG for L2 may reflect a distinct developmental trajectory than for L1.

Cerda et al. (2024) used this same cross-modal auditory multiplication task with proficient bilinguals and also observed an atypical IFG activation pattern between simpler and more difficult problems. Left IFG was engaged to a greater extent for small problems (i.e., problems with solutions < 25) than less rehearsed large problems (i.e., problems with solutions > 25), independent of the language of the operands. This is inconsistent with observed increases in IFG with more effortful processing. We proposed that the increased demand for hearing the problems in the cross-modal auditory task might have contributed to the reliance on more effortful retrieval even for small problems that are typically more rehearsed during early learning. Similarly, the current sample of adult L2 learners may have been especially taxed by the cross-modal paradigm in a weak language, rendering them unable to engage controlled retrieval processes for arithmetic when they are asked to verify problems presented in their L2.

This explanation would be consistent with findings from monolingual individuals with low and high math ability (Artemenko et al., 2019). Individuals with lower math ability show less activation in the IFG (and other regions including the left supramarginal gyrus and superior temporal gyrus), specifically when solving complex compared to simple division problems. The difference in activation of the IFG was interpreted as reflecting greater use of arithmetic fact retrieval and more efficient processing of arithmetic complexity by individuals with high math ability compared to individuals with lower math ability. The authors noted that since the left IFG is also associated with verbal processing and working memory (see Liakakis et al., 2011), increased engagement of the IFG during complex arithmetic processing in individuals with higher math ability might reflect increased control, verbal rehearsal, and maintenance of decomposed calculation steps during the application of rules (e.g., Gruber et al., 2001; Jost et al., 2009). By analogy, the reduced engagement of IFG for L2 problems in the current study may reflect a similar lack of engagement of more controlled processing as was observed in individuals with lower math skills.

Our results may also reflect the general inability of L2 learners to engage phonological or semantic control processes in their L2, particularly when they show low levels of expertise in this language. For the multiplication task, we observed a main effect of Language (i.e., [English-control] > [Spanish-control]) within the combined functional-anatomical mask of IFG, where significant clusters of activation fell entirely within the pars Triangularis. In a recent meta-analysis exploring the general organization of the language network, the pars Triangularis, along with the pars Opercularis, was associated with processes related to semantic and phonological control (Hodgson et al., 2021). In this context, semantic control was defined as the contextual selection and manipulation of semantic information necessary for task-appropriate behavior, particularly when dominant features must be suppressed, or subordinate meanings must be accessed (Badre et al., 2005; Davey et al., 2016; Jefferies, 2013). Previous research on bilinguals with fluent aphasia has also shown that these language control processes can be impaired asymmetrically across languages (e.g., Calabria et al., 2019). Similarly, our results may suggest that these processes could be differentially engaged in late L2 learners with limited L2 fluency.

In addition to predictions for the IFG and temporal regions, we predicted that verifying multiplication problems in L2 would engage the IPS more than L1, based on the assumption that L2 would depend less on direct retrieval processes. Although there was no significant difference in IPS engagement across languages across all problem types, an exploratory analysis of just true problems (removing false solutions, like 2 × 4 = 9) revealed that L2 (> control) engaged left IPS *less than* L1 (> control). This suggests that at least during verification of true multiplication problems, a weaker language may not engage numerical quantity processes that are typically associated with bilateral IPS (De Smedt et al., 2011; Prado et al., 2013). Alternatively, some have argued that at least during multiplication fact production, participants must access semantic memory for multiplication tables stored in the area lying along the IPS (Kazui et al., 2000). Under this interpretation, true problems presented in L2 may engage these numerical memory stores to a lesser extent than L1.

It is also possible that the relative deactivation that we observed for the IFG and IPS may be related to contrasting each language by the control condition of our task, in which participants were asked to press a button with their index finger when a blue box turned red. This condition was designed to subtract out any brain activity related to processing visual information at the solution and motor activity related to button press responses. In an exploratory analysis in which we contrasted the languages directly without the inclusion of the control conditions, we did not observe significant language differences in the left IFG or any of the other ROIs. However, this analysis revealed another potential source of differential processing across languages. At the whole brain level, we observed that English multiplications engaged the left middle occipital gyrus and the right inferior occipital gyrus to a greater extent than Spanish problems. The cluster on the left hemisphere encompassed the left Fusiform gyrus. Some have argued that the left and right fusiform gyrus support the visual form of Arabic digits (Dehaene et al., 2004; Yeo et al., 2017). The current study used a cross-modal task in which participants heard two operands and verified the correctness of an Arabic digit solution. Previous bilingual arithmetic studies have speculated that participants completing similar cross-modal tasks may be quickly transcoding the auditory stimuli into the digit format in preparation for verifying the Arabic digit solutions (Cerda et al., 2019, 2022, 2023; however, to date, this idea has not been directly tested. It is possible that L2 learners relied on transcoding processes from their L1 to the Arabic digit representation, resulting in more efficient processing (indicated by faster, more accurate responses) of multiplication problems in L1 to a greater extent than for their L2. This idea would be in line with a key prediction of the ECM (Campbell, 1994; Campbell & Clark, 1992; Campbell & Epp, 2004; Campbell et al., 1999) stating that because arithmetic is most frequently encountered in the Arabic digit format, it is the most efficient format for processing arithmetic.

In brief, contrary to predictions based on the limited literature on bilingual arithmetic, our sample of adult L2 learners showed a main effect of Language in IFG and IPS, but not temporal areas, with decreased engagement for L2 than L1. Robust behavioral differences were observed with slower and less accurate responses in L2 than L1. Reduced IFG and IPS engagement for L2 compared to L1 may reflect weaker engagement of effortful arithmetic retrieval processes similar to individuals with low math proficiency. Additionally, engagement of occipital regions for L1 greater than L2 may suggest that L2 learners access Arabic digit representations of the problems more in the L1 than in their L2.

### The Problem Size Effect in Second Language Learners

The L2 learners in the current study showed a typical Problem Size effect in their performance measures, with faster and more accurate responses to small problems (problems with correct solutions < 25) than large problems (problems with correct solutions > 25). The source of this behavioral effect remains debated (see Zbrodoff & Logan, 2005, for review). Some argue that performance differences stem from differences in memory retrieval demands for smaller, more practiced problems in comparison to less frequently encountered large problems. Campbell’s network interference hypothesis (Campbell, 1987; Campbell & Graham, 1985) suggests that because large problems have weaker associations between problem operands and their correct solutions in memory, retrieval of large problems involves increased interference from competing associations. In contrast, others argue that individuals tend to rely on entirely different strategies for small and large problems, with small problems relying on direct memory retrieval and large problems engaging more effortful decomposition or counting strategies (Campbell & Fugelsang, 2001; Campbell & Timm, 2000; Campbell & Xue, 2001; Hecht, 1999, 2002; LeFevre, Bisanz, et al., 1996; LeFevre, Sadesky, et al., 1996; Siegler, 1987). Our behavioral findings alone cannot address this debate directly, but our MRI results do provide additional insight into the potential source of these effects.

We found evidence that STG/MTG was engaged to a greater extent for small problems in comparison to large problems. This finding is in line with previous work showing increased activity in temporal regions for more practiced small problems, reflecting the strengthening of semantic associations between operands and their solutions (Prado et al., 2014). No significant voxels were observed within our regions of interest when plotting small problems > control and large problems > control. However, when plotting the mean activation values for these contrasts, it is apparent that the main effect of Problem Size in the STG/MTG was driven by deactivation for large problems (see Figure 7B). Van Rinsveld et al. (2017) have argued that temporal deactivation in response to simple arithmetic tasks may reflect less reliance on direct semantic associations between problems and solutions. Taken together, our results seem in line with Campbell’s network interference hypothesis, where large problems may have weaker semantic associations between problem operands and correct solutions.

The main effect of Problem Size in temporal regions fell primarily within the superior temporal gyrus (STG; see Figure 7B). According to Hodgson et al. (2021), the bilateral superior temporal gyri are thought to underpin phonological representations. Thus, the deactivation for large problems observed within STG might also reflect less engagement of phonological representations of large multiplication facts. In an exploratory whole brain analysis contrasting small and large problems, we found activation of additional regions perhaps supporting phonological processes (see Hodgson et al., 2021), including activation extending from left IFG to the left precentral gyrus and the right STG. The greater engagement of these phonological processes for small problems may be due to these problems being taught first, being more verbally rehearsed, and more frequently encountered in textbooks (Ashcraft & Christy, 1995).

Another common explanation for the Problem Size effect is that large problems are more likely to engage quantity-based strategies than direct retrieval (Campbell & Fugelsang, 2001; Campbell & Timm, 2000; Campbell & Xue, 2001; Hecht, 1999, 2002; LeFevre, Bisanz, et al., 1996; LeFevre, Sadesky, et al., 1996; Siegler, 1987). Thus, we hypothesized that the left IFG and bilateral IPS would be engaged to a greater extent for large problems in comparison to small problems, reflecting more effortful retrieval of less practiced arithmetic facts or involvement of quantity-based processes. However, our findings within our regions of interest were inconsistent with these hypotheses, revealing less engagement of both the left IFG and left IPS for large problems compared to small problems.

The main effect of Problem Size we observed in left IFG is consistent with a previous study in balanced bilingual adults performing an identical multiplication verification task, where the IFG was unexpectedly more active for small problems than large problems (Cerda et al., 2024). As noted above, we proposed that the increased demand for hearing the multiplication problems in our task might have contributed to the reliance on more effortful retrieval even for small problems. The cross-modal format of our task may have contributed to less efficient processing overall. In line with this idea, Finke et al. (2021) found that cross-format integration of numerical information from different symbolic formats occurs less rapidly (less efficiently) than within-format integration.

In contrast to the balanced bilinguals in Cerda et al. (2024), the L2 learners in the current study engaged the left IPS to a lesser extent for large problems compared to small problems. This result mirrored our exploratory finding that true problems in L2 engaged left IPS to a lesser extent than true problems in L1. Based on our original predictions, this might suggest that our L2 learners may not engage numerical quantity processes for large problems. However, in an exploratory analysis of the whole brain including only true solutions, we found that the left inferior parietal lobule was engaged to a greater extent for large problems than small problems. The left inferior parietal lobule is part of the system responsible for representation of numerical magnitude (see Suárez-Pellicioni et al., 2022). Thus, it is possible that L2 learners are still engaging quantity mechanisms for large problems, only when the problems are presented with their correct solutions. This same exploratory analysis also revealed that large problems engaged the right supramarginal gyrus to a greater extent than small problems. This region has been thought to be involved in the interaction between verbalization and visuospatial cognition during mental arithmetic (Houdé & Tzourio-Mazoyer, 2003; Zago et al., 2001). Thus, it is possible that at least when large problems are presented with their correct solutions, L2 learners rely on processes related to quantity mechanisms and verbalization of numerical information.

In sum, we observed mostly expected Problem Size effect pattern in our behavioral and brain data, based on previous studies of bilingual arithmetic. Our sample of adult L2 learners engaged STG/MTG and IFG to a greater extent for small problems, indicating increased reliance on verbal retrieval processes for those problems. Although it was expected that large problems would engage left IPS to a greater extent than small problems, large problems engaged left IPS to a lesser extent than small problems. However, we still found evidence that L2 learners still engage quantity mechanisms for large problems, but only when the problems are presented with their correct solutions. This may suggest that problems with incorrect solutions may drive differential processing of larger, more difficult problems.

### Interactions Between Language and Problem Size

We had expected a significant interaction between Language and Problem Size given previous work with both monolinguals (Dickson & Wicha, 2019) and bilinguals (Cerda et al., 2023), where less familiar formats (or languages) promoted inefficient processing particularly for larger, less practiced problems. Based on the ECM, which argues for language-specific arithmetic memory representations (Campbell, 1994; Campbell & Clark, 1992; Campbell & Epp, 2004; Campbell et al., 1999), L2 learners with limited L2 experience should have a separate, weaker memory network for arithmetic in their L2, where associations between arithmetic problems and their solutions is expected to be less robust. Thus, retrieval of large problems in L2 should have been less efficient than L1, given that the representation of large problems in L2 should be weakest in memory. At the behavioral level, we predicted that this would be reflected by significantly slower response times and/or significantly reduced accuracy for large problems in L2 compared to small ones.

Our behavioral data did reveal an interaction between language and Problem Size only for response time, where the Problem Size effect was larger for L1 than L2. This interaction remained significant in our analysis of only true problems. However, the pattern of results revealed that the difference between small problems across languages was larger than the difference between large problems across languages (average language difference for small problems = 151.3 ms; average language difference for large problems = 84.4 ms). According to the predictions of the ECM, we expected a larger language difference for large problems, since large problems in L2 should be less efficiently accessed from memory than any other problem type. In a seminal study on bilingual arithmetic, Marsh and Maki (1976) hypothesized that if bilinguals were accessing language-specific representations for arithmetic, then it should take progressively longer to respond in the nonpreferred language relative to the preferred language as problems increase in complexity (i.e., a significant interaction between Language and Problem Size, driven by increased response times for more difficult problems). Instead, we observed the opposite pattern for our L2 learners, whereas problems increased in difficulty, language differences were not as large in comparison to simpler, small problems. Our results suggest that L2 learners have increased familiarity with over-rehearsed small problems in their L1 in comparison to their L2, especially since we did not observe any interactions between language and Problem Size for accuracy. Indeed, as mentioned above, small problems are more likely to be taught first, more verbally rehearsed, and more frequently encountered in textbooks (Ashcraft & Christy, 1995), and our population only rehearsed their facts in L1 during early learning. This may also be related to potential transcoding of problems in L1 to an Arabic digit format (see above). Critically, the interaction we observed did not seem to result from a compounding difference as problems increased in difficulty.

Marsh and Maki (1976) also did not find evidence that bilinguals took progressively longer to solve more difficult problems. In their study, they asked Spanish–English bilinguals who ranged in language fluency to add or subtract a series of single Arabic digits and produce a response in their preferred or non-preferred language for arithmetic. Problems varied in complexity, involving either 1, 2, or 3 operations. In contrast to the current results, they observed no evidence of an interaction between problem complexity and language. Instead, they found that participants were only faster overall when responding to problems in their preferred language. They proposed two possible explanations for their results: (1) Overall differences in response time might reflect the time required to translate from the non-preferred language to access the representation of the information in the preferred language or (2) Both languages must be translated from an abstract representation, where the non-preferred language simply takes longer. Either explanation suggests a single representation for both languages, where additional processing time in an L2 reflects processes related to some form of translation. Given that we did find an interaction between Language and Problem Size for response time, it is possible that L2 learners may be accessing language-specific representations for arithmetic, where small problems have more efficient problem-solution associations for L1. However, our imaging results do not support this idea.

In terms of brain activation, we may have expected a significant Language by Problem Size interaction within STG/MTG, as these temporal regions are thought to be involved in the storage of arithmetic facts in verbal memory, and have been shown to be differentially engaged based on the strength of semantic associations between problems and solutions (Van Rinsveld et al., 2017). However, we did not observe any interactions between Language and Problem Size for measures of brain activation. Thus, at least in our imaging data, we did not find evidence in support of math cognition models that argue for language-specific representations of arithmetic facts, where L2 would be expected to engage a separate, weaker arithmetic memory representation (i.e., ECM; Campbell, 1994; Campbell & Clark, 1992; Campbell & Epp, 2004; Campbell et al., 1999). Taken together with the main effects of language we observed, it seems that language differences in performance may reflect weaker engagement of effortful arithmetic retrieval processes in L2 similar to individuals with low math proficiency. Additionally, engagement of occipital regions for L1 over L2 may suggest access to Arabic digit representations of the problems in the L1, perhaps especially for small problems as the most rehearsed problem type.

### CONCLUSIONS

We found that L2 learners verified single-digit multiplication problems faster and more accurately in their L1 compared to their L2. We also observed an interaction between Language and Problem Size for response time, where easier problems in L1 were responded to more quickly than any other problem type. The imaging data suggests that the language differences in performance were not driven by differences in recruitment of the storage of arithmetic facts, as we did not observe expected differences in the engagement of the superior and middle temporal gyri (STG/MTG) across languages. Surprisingly, we found that late L2 learners engaged the left IFG to a lesser extent for L2 than L1. This could suggest that language differences in behavior may have been driven by their ability to engage more effortful arithmetic retrieval processes in L1, but not in their weaker language, L2. Supporting this idea, individuals with lower math ability engage IFG to a lesser extent than individuals with higher math ability, suggesting that individuals with higher math ability exhibit increased control, verbal rehearsal, and/or maintenance of decomposed calculation steps during arithmetic processing. Additionally, L2 learners engaged visual processes to a greater extent in their L1 than L2, suggesting that they also may have been relying on the Arabic digit representation of arithmetic facts when processing multiplication in L1.

The adults in the current study verified small problems faster and more accurately than large problems, a typical pattern based on Problem Size. They engaged the left STG/MTG, left IFG, and the left IPS to a lesser extent for large problems compared to small problems. In line with previous studies, the Problem Size effect observed in temporal regions supports the idea that large problems engage phonological representations of multiplication facts to a lesser extent than small problems. Although the observed Problem Size pattern in the left IFG may be inconsistent with previous neuroimaging studies in monolinguals, the pattern is consistent with a previous bilingual study using an identical auditory multiplication verification task. This further suggests that the increased demand for hearing the problems might have contributed to the reliance on more effortful retrieval even for small multiplication problems. Additionally, although we did not observe expected effects of Problem Size within bilateral IPS, we did find evidence that large problems do engage numerical quantity processes, at least when problems were presented with their correct solutions.

Overall, our imaging results do not support the idea that entirely separate arithmetic memory networks exist in L1 and L2, as proposed by the ECM. Based on the ECM, retrieval of large problems in L2 should have been less efficient than L1, given that the representation of large problems in L2 should be weakest in memory. However, we did not observe any interactions between Language and Problem Size in measures of brain activation supporting this prediction. Although we did observe a Language by Problem Size interaction for response time on our task, the interaction seemed to be driven by increased efficiency of small problems in L1 rather than increased inefficiency of processing more difficult problems in L2. The TCM does not directly address bilingualism but suggests that L2 learners should engage direct retrieval for arithmetic facts in L1 and would need to translate into L1 when problems are presented in L2. We did not find evidence that L2 learners differentially engaged the arithmetic representations housed in STG/MTG across languages, as we only observed differences in engaging effortful retrieval processes across languages. It is possible that L2 learners relied on transcoding or translation processes from each language to the Arabic digit representation (or another abstract numerical representation) to verify simple multiplication problems. Future research is needed to directly test this idea.

## ACKNOWLEDGMENTS

This work was performed using instruments housed in the Vanderbilt Center for Human Imaging. We thank Megan Garcia for assistance with participant recruitment, data collection, and data organization. Lastly, we thank all the L2 learners for their time and participation in this study.

## FUNDING INFORMATION

Nicole Y. Wicha, Foundation for the National Institutes of Health (https://dx.doi.org/10.13039/100000009), Award ID: 5R21HD098878. Vanessa R. Cerda, Foundation for the National Institutes of Health (https://dx.doi.org/10.13039/100000009), Award ID: 8K00HD112280.

## AUTHOR CONTRIBUTIONS

**Vanessa R. Cerda**: Conceptualization: Equal; Formal analysis: Lead; Investigation: Lead; Visualization: Lead; Writing – original draft: Lead; Writing – review & editing: Lead. **Macarena Suárez-Pellicioni**: Conceptualization: Supporting; Methodology: Supporting; Writing – review & editing: Supporting. **Nicole Y. Wicha**: Conceptualization: Equal; Funding acquisition: Lead; Methodology: Lead; Resources: Supporting; Writing – review & editing: Supporting. **James R. Booth**: Conceptualization: Supporting; Formal analysis: Supporting; Funding acquisition: Supporting; Investigation: Supporting; Project administration: Lead; Resources: Lead; Software: Lead; Supervision: Lead; Writing – review & editing: Supporting.

## DATA AND CODE AVAILABILITY STATEMENTS

All analyses were preregistered on Open Science Framework (OSF) (see https://osf.io/wfgvk). Additionally, all data and code used are shared on the associated project folder on OSF (https://osf.io/z9ajt).

## Notes

^1^ This analysis was added per reviewer request.^2^ Note that the behavioral data was previously analyzed using linear mixed models, including participant averages of within-subject repeated measures of accuracy and response time for each condition. Per reviewer request, we updated this analysis to consider trial-level accuracy and response time data. Note that our reported results did change slightly. For accuracy, the main effect of Correctness and the interaction between Problem Size and Correctness was significant in the original analysis, but these effects were no longer significant in the updated analysis. Additionally, in the original analysis of response time, there were no significant interactions between the factors of interest. However, the updated analysis revealed a significant interaction between Language and Problem Size.^3^ This analysis was added per reviewer request.^4^ These exploratory maps were added, per reviewer request.^5^ This analysis was added, per reviewer request.

## TECHNICAL TERMS

Encoding Complex Model: The *Encoding Complex Model* is a leading model of arithmetic processing that suggests that separate arithmetic memory representations exist for each format in which problems appear, where retrieval efficiency is dependent on experience using the facts in each format.
Problem Size Effect: The *Problem Size Effect* is a well-known phenomenon in the math cognition literature, where small problems (e.g., 2 × 3) are typically more practiced and are retrieved more efficiently than large problems (e.g., 8 × 9).
Balanced bilinguals: *Balanced bilinguals* are bilinguals who have relatively equivalent languages in each of the languages they speak.
